# A Multimodal Approach towards Genomic Identification of Protein Inhibitors of Uracil-DNA Glycosylase

**DOI:** 10.3390/v15061348

**Published:** 2023-06-10

**Authors:** Wael Muselmani, Naail Kashif-Khan, Claire Bagnéris, Rosalia Santangelo, Mark A. Williams, Renos Savva

**Affiliations:** Institute of Structural and Molecular Biology, Department of Biological Sciences, Birkbeck, University of London, Malet Street, London WC1E 7HX, UKma.williams@bbk.ac.uk (M.A.W.)

**Keywords:** virus, bacteriophage, MRSA, SCCmec, uracil-DNA glycosylase, Ugi

## Abstract

DNA-mimicking proteins encoded by viruses can modulate processes such as innate cellular immunity. An example is Ung-family uracil-DNA glycosylase inhibition, which prevents Ung-mediated degradation via the stoichiometric protein blockade of the Ung DNA-binding cleft. This is significant where uracil-DNA is a key determinant in the replication and distribution of virus genomes. Unrelated protein folds support a common physicochemical spatial strategy for Ung inhibition, characterised by pronounced sequence plasticity within the diverse fold families. That, and the fact that relatively few template sequences are biochemically verified to encode Ung inhibitor proteins, presents a barrier to the straightforward identification of Ung inhibitors in genomic sequences. In this study, distant homologs of known Ung inhibitors were characterised via structural biology and structure prediction methods. A recombinant cellular survival assay and in vitro biochemical assay were used to screen distant variants and mutants to further explore tolerated sequence plasticity in motifs supporting Ung inhibition. The resulting validated sequence repertoire defines an expanded set of heuristic sequence and biophysical signatures shared by known Ung inhibitor proteins. A computational search of genome database sequences and the results of recombinant tests of selected output sequences obtained are presented here.

## 1. Introduction

The pyrimidine-base, uracil, occurs frequently in DNA under ambient cellular conditions: In a mammalian cell, approximately 100–500 cytosine bases per day will deaminate to form U:G mismatches, and around 10^4^ misincorporations of dUTP (a natural precursor for thymidine biosynthesis) will occur in each round of replication to create U:A base pairs [1]. Assuming rates are comparable in bacterial cells, hundreds of dUTP misincorporations would occur per round of replication, and on the order of 1 in 250 cytosine positions would deaminate per day. Perturbed cellular function could be expected unless uracil is removed and replaced with the canonical base. For example, thymine methyl groups are important chemical signatures for DNA binding proteins such as transcription factors during motif recognition [2], while U:G base pairs would transition to T:A during the replication of the affected DNA strand.

When viruses replicate in a cell, the nucleotide pools are imbalanced to the extent that the further misincorporation of uracil would be inevitable [3]. Some viruses, particularly bacteriophages, manage uracil-DNA via specialised viral genome encoded factors, either by utilising it as a protective strategy against restriction enzymes or by suppressing it if it interferes with the replication strategy [4,5,6,7].

Bacterial cellular dUTPase will minimise the misincorporation of dUTP, and the host’s uracil-DNA glycosylase (UDG) will remove uracil from DNA [8]. Ung (family 1 of the UDG superfamily) is the most prevalent form of UDG and removes uracil bases, regardless of their state of base pairing or sequence context, via the cleavage of the glycosyl bond attaching them to the deoxyribose [9]. Unless it is enzymatically processed, the abasic site created by Ung is relatively stable under ambient cellular conditions [10].

For DNA repair purposes, UDG/Ung initiates a process known as base excision repair (BER), in which a specialised DNA endonuclease (endonuclease IV in bacterial cells) will rapidly recognise and cleave the DNA backbone and under ordinary circumstances would recruit a specialised repair DNA polymerase (PolA in bacterial cells); repair is then completed by a DNA ligase (LigA in bacterial cells) [11,12].

However, in the presence of replicating viral DNA, where millions of copies accumulate over so-called “burst“ periods (measured in minutes), Ung is in effect a restriction factor. Since Ung promotes endonuclease cleavage of the DNA backbone, double-stranded viral DNA will fragment if uracil bases appear in close proximity [13]. This DNA fragmentation will also affect viruses whose DNA replication involves programmed backbone nicks or exposed single-stranded DNA regions, and transposable elements where the translocation of naked single-stranded DNA is necessary during conjugative transfer.

To mitigate against Ung restriction effects, bacteriophages may encode protective factors, such as a dUTPase or uracil-DNA glycosylase inhibitors [7,14,15]. The latter are presently known to target Ung specifically. Ung inhibitors (which are interchangeably referred to herein as UngIns) are DNA mimetic proteins utilising charge-based alignment with the Ung DNA binding cleft to dock Ung. Docking promotes the hydrophobic sequestration of the sidechain of the apical residue in the Ung DNA minor groove binding loop, which is essentially irreversible under biological conditions, to inhibit Ung [16,17].

So effective is the strategy of Ung inhibition that under its protection, some phages are known to encode entire nucleotide biosynthesis pathways with specialised DNA and RNA polymerases to replicate their genomes in the form of thymine-free uracil-DNA, thereby avoiding sensitivity to host restriction endonucleases [18,19,20,21,22,23].

UngIn protein structures described to date fall into one of three observed folds: Ugi/SAUGI (note that Ugi and SAUGI share the same protein fold at ~12% sequence residue identity), p56, and Vpr; yet, within each fold, type there is pronounced sequence plasticity, to the extent that the unambiguous identification of an Ung inhibitor in any genome is rarely straightforward. In some organisms using uracil in their own genomes, such as the uracil-DNA Yersinia phage PhiR1-37 [21], the identification of an encoded UngIn has proven to be impossible with common bioinformatics tools.

It is possible that other forms of Ung modulation could be deployed, such as that described for Escherichia phage T5 [24], or that some UngIn encoding sequences could be beyond our present limits of detection. These undetectable sequences may belong to as-yet unknown, structurally diverse families.

At best, novel UngIns may be discovered through homology to known inhibitor sequences, their known genomic loci, or through predicted biophysical properties of gene products; however, such methods have not thus far permitted UngIn identification in genomes such as *Yersinia* phage PhiR1-37.

The prototypical known UngIn families (Ugi, SAUGI, and p56) display significant sequence diversity. Sequence identities as low as 20% are typical, which makes the computational identification of UngIns difficult in the absence of experimental data [16]. This poor sequence conservation can lead to the misannotation of UngIn sequences. Not all such sequences in genome records are annotated as uracil-DNA glycosylase inhibitors, and even when they are thus annotated, it cannot be taken for granted that these proteins are viable or act as functional UngIns.

For these reasons, distant homologs of each UngIn type (Ugi, SAUGI, and p56) were selected for the experimental screening of UngIn activity. Heuristic signatures of UngIn sequences were defined from verified UngIn variants and subsequently used to search for UngIns in virus or other pathogen genomes.

In this study, the aim is to develop an expanded, validated sequence repertoire for UngIns, based upon structural insights and directed random mutagenesis, and use it to search phage genomes for uracil-DNA glycosylase inhibitors. Genomic sequences are searched using heuristic signatures that essentially define the properties of currently known Ung inhibitors, and the output sequences are assessed via recombinant expression and UngIn function assays.

## 2. Materials and Methods

### 2.1. In Silico Identification of Potential Homologs of Ugi

A PSI-BLAST [25] search was performed online (NCBI) on the non-redundant protein sequences database using the Ugi sequence (Accession: YP_009664501.1) from *Bacillus* phages PBS1 as a template (note that *Bacillus* phages PBS1, PBS2, and AR9 encode identical Ugi protein sequences). Default search parameters were used. Iterations were run until no new sequences below the E-value threshold (0.05) were obtained.

### 2.2. In Silico Identification of Potential Homologs of SAUGI

A PSI-BLAST [25] search was performed online (NCBI) on the non-redundant protein sequences database using the SAUGI sequence (UniProtKB-Q936H5_STAAU) as a template. Default search parameters were used, with maximum target sequences set at 20,000. Iterations were run until no new sequences below the E-value threshold (0.05) were obtained. After each iteration, all the sequences below the E-value threshold and selected sequences above the E-value threshold (satisfying the condition: query cover > 80% and percent identity > 25%) were included to build the position-specific scoring matrix (PSSM) for the next iteration.

Hits generated from this procedure that were encoded by species other than *Staphylococcus* with >80% query cover and <35% percent identity were further investigated as potential distant homologs of SAUGI.

### 2.3. In Silico Identification of Potential Homologs of p56

An online PHI-BLAST [26] search at the NCBI was performed using the p56 sequence (Accession: NP_040721.1) encoded by *Bacillus* phage PZA as a template and E-X(2)-Y-X(0,2) G as a PROSITE pattern. The search was iterated until no new sequences were obtained. Hits generated from this procedure, found to be encoded by phages, were further investigated as potential p56 homologs.

Additionally, a PSI-BLAST search was performed on the non-redundant protein sequences database, selecting the *Salasmaviridae* organism dropdown (p56 is known to be encoded by *Salasmaviridae* phages). The search was iterated until no new sequences were obtained.

### 2.4. Heuristics Analyses of Ugi and SAUGI Sequences

Sequences generated from the SAUGI PSI-BLAST search with >80% query cover, and the sequences generated from the Ugi PSI-BLAST search were aligned using Clustal Omega [27] or MUSCLE [28] as part of the MEGA X suite [29], or MAFFT [30]. Sequence logos were made using Weblogo 3 [31].

### 2.5. Phage Genome Database Filtering

Filter scripts were written in the VSCode IDE [32] in Python using tools from the Biopython module [33]. Scripts were run on individual genomes or bulk genome lists, downloaded from the NCBI database [34]. Genomes were initially translated into all six reading frames, with any continuous sequence between two stop codons treated as a putative polypeptide (with a minimum length of 40 amino acids). Any N-terminal sequence before the first valid start codon in each sequence was removed—start codons were defined as M, I, V, or L [35]. While there is evidence of additional start codon usage in bacteria [36], these were excluded in the interests of stringency in sequence processing.

Processed sequences were then passed through a set of filters that were defined based on the heuristics analysis, with sequences passing each filter check written to an output FASTA file. For bulk genome inputs, additional filtering and binning was applied—genomes were binned based on GC content and screened for additional motifs. Parameters for each filter were optimized such that the known UngIn sequence was returned in the final output, along with as few false positive sequences as possible. Scripts and data are available at https://github.com/naailkhan28/ung_inhibitor_heuristics (5 June 2023).

### 2.6. Cloning of Candidate Genes

Enzymes and bacterial strains used in this study were obtained from New England BioLabs (NEB, Ipswich, MA, USA); synthetic DNA was sourced from Integrated DNA Technologies (IDT, Coralville, IA, USA) or Sigma Aldrich (Waltham, MA, USA), as indicated. Site-directed mutagenesis was performed using the Q5^®^ Site-Directed Mutagenesis Kit (NEB). Purification of DNA from enzymatic reactions and from agarose gel slices was achieved using the QIAquick gel extraction kit (QIAGEN, Hilden, Germany).

Coding sequences for candidate Ung inhibitory genes were optimised for recombinant expression in *E. coli* via the manual adjustment of codon usage following initial analysis using the *E. coli* Codon Usage Analyzer 2.1 tool by Morris Maduro [37]. Any sequential codons considered sub-optimal were replaced with silent alternatives to limit local concentrations of sub-optimal codons from the 11th codon onwards to no more than 1 in any 5-codon window and no more than 2 in total per 105-nucleotides of sequence. Subsequent minimal silent manual adjustment to remove or insert restriction enzyme recognition sites was performed using NEBcutter V2.0 [38]. Whilst maintaining earlier editing aims, further minimal manual silent adjustments were made to limit homo-polynucleotide runs and predicted mRNA hairpins to <7 nucleotides in length; these were performed guided by output from the UNAFold Web Server [39]. Synthetic desiccated gBlocks^TM^ Gene Fragments (IDT) were resuspended in autoclaved reverse osmosed deionised water to a concentration of 1 ng/µL.

Amplicons of target open reading frames (ORFs) were generated from this template DNA via PCR with Q5^®^ High-Fidelity DNA Polymerase (NEB). PCR primers (Sigma-Aldrich, or IDT) incorporated overlap complementarity with the pRSET-C expression vector (Invitrogen, Waltham, MA, USA). A linear amplicon of pRSET-C was created by inverse PCR using Q5^®^ High-Fidelity DNA Polymerase. ORF amplicons and the linear vector amplicon were gel purified from 1% TAE-agarose using a QIAquick gel extraction kit (QIAGEN). Primers for overlap extension PCR were pre-phosphorylated using T4 PNK (NEB). Linear precursors of expression constructs were formed via overlap extension PCR (OE-PCR) using 2 ng of each of the respective purified DNA. OE-PCR amplicons were ligated using T4 DNA ligase (NEB) and propagated in NEB^®^ 5-alpha cells. SupremeRun Sanger sequencing services from GATC were used to verify constructs for expression analysis (Eurofins Genomics, Louisville, KY, USA).

### 2.7. Expression of Candidate Genes

T7 Express *lysY/I^q^* competent *E. coli* cells were transformed with the relevant expression vector. To perform small-scale expression, a single colony from each transformation plate was transferred aseptically to sterile 5 mL LB media containing 100 µg/mL ampicillin and 2% (*w*/*v*) α-D-glucose in a sterile 30 mL universal tube. Inoculated tubes were incubated at 37 °C with shaking at 220 rpm until a cell density of 0.6–0.8 was reached, as measured via spectrophotometric absorbance at 600 nm. Culture tubes were moved to a water-ice bath for 2 min. To induce recombinant expression, filter-sterilised 1 M isopropyl β-D-1-thiogalactopyranoside (IPTG) was added to cultures for a final concentration of 0.5 mM. Tubes were then swirled and placed in a 37 °C water bath for 2 min, prior to their return to a shaker incubator at the required temperature for expression. Induced cells were incubated with shaking for a further 12–16 h at 18 °C. Absorbance was measured at 600 nm (OD_600_), and cells were harvested by centrifugation at 4 °C for 20 min at 4500× *g* and stored at −20 °C for later use. Pellets were resuspended in a volume of lysis buffer equal to 1/8 OD_600_ for each sample aliquot of cell culture; the utilised lysis buffer (25 mM Tris, 50 mM NaCl, pH 8) included 1× Protease Inhibitor Cocktail Tablet solution (Roche, Basel, Switzerland). Sonication of 0.5 mL aliquots from resuspended samples was performed using a Sonics Vibra-cell ultrasonic processor, for 2 min, 60 W amplitude, with 3-s on/off pulses. The resulting lysate was centrifuged at 18,000× *g* for 30 min at 4 °C, and the supernatant was decanted for storage at −20 °C to later visualise the soluble fraction of the cell in SDS-PAGE, or at 4 °C to be used for UDG assay within 30 min.

Large-scale expression was performed in 2 L capacity plain Erlenmeyer flasks, each containing 500 mL sterile LB media (autoclaved 30 min at 121 °C and 15 psi). Filter-sterilised (0.22 μm) ampicillin at 100 µg/mL and α-D-glucose at 2% (*w*/*v*) were added to the autoclaved flasks. The inoculation of the flasks proceeded via cells from 5 mL log phase cultures, harvested and resuspended in 5 mL fresh sterile (autoclaved) LB media before addition at 1/100 (*v*/*v*) ratio to the flasks.

### 2.8. Protein Purification

For purification from 5 mL culture volumes, stepwise fractionation of the soluble fraction from cell lysates was performed in centrifugal filter units of 5.0 µm pore-size filled with 400 µL of Q-Sepharose™ Fast Flow (Amersham Biosciences, Amersham, UK). The filter unit resin bed was equilibrated using 4 mL of a low-salt concentration buffer (50 mM NaCl, 20 mM Tris, pH 8.0). The soluble fraction of lysed cells was loaded onto the equilibrated Q-Sepharose, and then the filter units were gently flicked and inverted 5 to 6 times before letting the resin bed set for 2 min. The filter units were spun down at 300× *g* for 2 min. Elution proceeded via the stepwise application of buffers containing incremental amounts of salt (Buffers 1 to 11; Supplementary Table S1). In each step, 250 µL of buffer was added; note: buffers 1 and 11 were each, respectively, added three steps in succession. Filter units were centrifuged at 300× *g* at each step for 2 min. Eluted fractions were analysed by SDS-PAGE to identify fractions with >90% protein purity.

Buffers used for large-scale purification are listed in Supplementary Table S2. For purifications from large-scale cultures, the obtained cell paste was resuspended in Buffer A at a 1/5 (*w*/*v*) ratio. Cells were lysed by sonication (parameters as described earlier) and purification was performed via sequential chromatographic steps.

The AKTA fast protein liquid chromatography (FPLC) system was used for all chromatography steps. Fractions corresponding to peaks in chromatography steps were analysed by SDS-PAGE, and fractions that contained post-induction protein consistent with the expected mass relative to a protein standard ladder were included in the next chromatography step.

For His-tagged proteins, the first chromatographic step involved immobilized metal affinity chromatography (IMAC), wherein a 1 mL HisTrap HP column (GE) was equilibrated with 20 column volumes of buffer B. The resuspended sample in buffer A was loaded on the equilibrated column. The column was then washed with 20 column volumes of buffer B. Samples were eluted by applying a 30-column volume linear gradient of buffer B to buffer C. The eluted fractions corresponding to absorbances significantly above baseline were collected and diluted with buffer F to achieve a final concentration of 50 mM NaCl.

In ion exchange chromatography (IEC), 1 mL HiTrap Q HP column (GE) was equilibrated with 20 column volumes of buffer D before loading the IMAC diluted sample. The column was then washed with 20 column volumes of buffer D. Samples were eluted by applying a 30-column volume linear gradient of buffers D to E. The eluted fractions corresponding to absorbances significantly above baseline were collected and concentrated at 3200× *g* to a final volume of 1 mL using an Ultracel 3K centrifugal filter (Millipore, Burlington, MA, UAS).

For size exclusion chromatography (SEC), the concentrated sample obtained from IEC was applied to a 120 mL Superdex 75 column previously washed with 1 column volume of double distilled water followed by equilibration with 3 column volumes of buffer G. Eluted fractions containing the pure target protein were concentrated at 3200× *g* using an Ultracel 3K centrifugal filter (Millipore) to reach the required protein concentration. For Ugi-2:SAUNG complex purification, Ugi-2 and SAUNG were expressed from constructs pRSCUgi-2 (a pRSET-C vector carrying the Ugi-2 insert) and pRS-SAUNG6xH (a pRSET-C carrying a C-terminal His-tagged SAUNG) separately at a large scale. Cell pellets were resuspended in buffer A; resuspended cells were mixed prior to performing cell lysis. The mixed cell extract was sequentially purified by IMAC, anion IEX, and, finally, SEC.

MCUGI1 was expressed and co-purified with SAUNG in the same manner as described for Ugi-2 with the difference that clarified lysates were mixed rather than resuspended cell pellets.

The VMY22 p56 was co-expressed and co-purified with *Bacillus Weidmannii* Ung (BwUng) analogously as previously described [17], with the difference of using a construct that carries VMY22 p56 and BwUng inserts in place of PZA p56 and HSV-1 UNG inserts, respectively (Supplementary File—Section S1).

### 2.9. Crystallisation of Ugi-2 in Complex with SAUNG

For the crystallisation of the Ugi-2 in complex with SAUNG, a purified protein sample was used at 15 mg/mL in commercial crystallisation screens (JCSG-Plus and PACT premier; Molecular Dimensions, Rotherham, UK) [40]. Sitting drops were prepared at both 1:1 and 2:1 ratios of protein to mother liquor with volumes of 75 nL and 100 nL protein, respectively; plates were incubated at 16 °C. Protein crystals were obtained from JCSG-Plus condition D5 (0.1 M HEPES, pH 7.5, 70% *v*/*v* MPD). These crystals exhibited a flat rectangular plate morphology and were flash-frozen in liquid nitrogen without adding cryoprotectant as the mother liquor of this condition includes MPD, which can act as a cryo-protectant.

### 2.10. Crystallisation of MCUGI1 in Complex with SAUNG

A purified protein sample was used at 25 mg/mL in commercial crystallisation screens (JCSG-Plus and PACT premier; Molecular Dimensions) [40]. Sitting drops were prepared at both 1:1 and 2:1 ratios of protein to mother liquor with volumes of 75 nL and 100 nL protein, respectively; plates were incubated at 16 °C. Protein crystals were obtained from JCSG-Plus condition E2 (2.0 M Ammonium sulphate, 0.2 M Sodium chloride, 0.1 M MES, pH 6.5). These crystals exhibited a sea urchin morphology. An optimised condition of 1.0 M ammonium sulphate, 0.2 M sodium chloride, 9.55% (*w*/*v*) PEG 20.000, and 0.1 MMES, at pH 6.5 led to the formation of better-diffracting needle-shaped crystals. These crystals were cryoprotected using the mother liquor solution supplemented with 25% (*v*/*v*) ethylene glycol.

### 2.11. Crystallisation of VMY22 p56 in Complex with BwUng

A purified protein sample was used at 14 mg/mL in commercial crystallisation screens (JCSG-Plus and PACT premier; Molecular Dimensions). Sitting drops were prepared at both 1:1 and 2:1 ratios of protein to mother liquor with volumes of 75 nL and 100 nL protein, respectively; plates were incubated at 4 °C. Protein crystals were obtained from PACT premier condition H4 (0.2 M Potassium thiocyanate, 0.1 M BisTris propane, pH 8.5, 20% (*w*/*v*) PEG3350). These crystals were cryoprotected using the mother liquor solution supplemented with 20% (*v*/*v*) glycerol.

### 2.12. Structure Determination

X-ray diffraction data was collected on beamline IO4 at the Diamond light source facility (Ugi-2 in complex with SAUNG, and VMY22 p56 in complex with BwUng) or beamline ID30B at the European Synchrotron Radiation Facility (MCUGI1 in complex with SAUNG). The xia2 pipeline was used for automated data processing using XDS; AIMLESS was used to scale the structure factors. The structures were initially phased by molecular replacement with MOLREP [41] using Chain A from PDB accession 3WDG (complexed to SAUNG) [42] and chain I from PDB accession 1UDI (complexed to PBS1 Ugi) [43] as search models for the structure of Ugi-2 in complex with SAUNG. Chain A from PDB accession 3WDG was used in molecular replacement as a search model for the structure of MCUGI1 in complex with SAUNG, and 4L5N (The complex of PZA-p56 and HSV-1 UNG) [17] as a search model for the structure of VMY22 p56 in complex with BwUng. Structure refinement used REFMAC5 [44] and model building was performed using the program COOT [45]. Statistics for data collection and refinement are summarised in Table 1.

### 2.13. Structure Predictions of Potential UngIn Homologs

Structure predictions for monomers were performed using AlphaFold2 [46]. Dimer and protein-complex structure predictions were performed using AlphaFold-Multimer [47]. All predictions were run using the Google Colab notebook ColabFold [48]. For each predicted structure, the model with the highest predicted local distance difference test (pLDDT) score was used for structural analysis.

### 2.14. Library Mutagenesis of Ugi

Inverse PCR of the pBUGI8 and pSDM4_Ugi_Ung plasmids was performed with oligonucleotides containing designed mutation propensities (Supplementary File—Section S1). Primers were phosphorylated with T4 polynucleotide kinase prior to use in a 20-cycle PCR performed using Q5^®^ High-Fidelity DNA Polymerase. Amplicons were purified following electrophoresis from 0.8% agarose and adjusted to 50 µL at 2 ng/µL for circularisation using T4 DNA ligase (NEB) at 30 °C for 2-h. NEB^®^ 5-alpha cells were transformed to propagate the ligation reaction.

### 2.15. In Vitro UDG Activity/Inhibition Assay

Ung inhibition was assayed in vitro using an agarose gel electrophoresis assay [17]. *Taq* PCR amplicons containing uracil-DNA were subjected to enzyme treatment with purified Ung, and serial dilution in STE buffer was used to discover the minimum enzyme concentration needed for the digestion of 5 µL of uracil-DNA substrate. The results were compared to tests with control PCR amplicons carried out without uracil.

Putative UngIns were screened for inhibitory activity, using 3 µL of purified protein from small-scale expression cultures, or 3 µL of the soluble fraction of cell lysates of these cultures, harvested 12–16 h post-induction.

Positive controls were previously purified, known as Ung inhibitors: Ugi from *Bacillus subtilis* phage PBS1 [49], SAUGI [42], and bacteriophage PZA p56 [17]. Cell lysates from plasmid-free T7 Express *lysY/I^q^* cells and from the *dut^−^/ung^−^*-deficient *E. coli* strain CJ236, were used as cell lysate controls.

### 2.16. In Vivo Ung-Inhibition Assay, Vector Construction

A high-copy-number vector, designated pBpST-CAT was created by the site-directed mutagenesis of the T7 promoter sequence of pRSET-C (Invitrogen) to a Trc promoter sequence (Supplementary File—Section S1).

The construct pBpST-CAT was linearised via a 1 h HindIII-HF/NdeI double digest at 37 °C in the presence of 0.5 units CIP alkaline phosphatase and purified following electrophoresis from 0.8% agarose. The Ugi gene was obtained from pBUGI8 [49] via a 1 h HindIII-HF/NdeI double digest at 37 °C and purified following electrophoresis from 1% agarose. The plasmid (100 ng) and insert were ligated for 2 h at 25 °C in a 1:3 molar ratio using T4 DNA ligase (NEB), and recombinants were isolated from clonal colonies of transformed NEB^®^ 5-alpha cells. The sequence-verified construct was designated pSDM4_Ugi.

The construct pSDM4_Ugi was linearised, via a 1 h BspEI digest at 37 °C in the presence of 0.5 units CIP alkaline phosphatase and purified following electrophoresis from 0.8% agarose. The HSV-1 UNG gene was obtained from pTS106.1 [50] via PCR using Q5^®^ High-Fidelity DNA Polymerase (NEB), (Supplementary File—Section S1). The amplicon was digested for 1 h in the presence of BspEI and AgeI at 37 °C and purified following electrophoresis from 1% agarose. The plasmid (100 ng) and insert were ligated for 2 h at 25 °C in a 1:3 molar ratio using T4 DNA ligase (NEB) and recombinants were isolated from clonal colonies of transformed NEB^®^ 5-alpha cells. The sequence-verified construct was designated pSDM4_Ugi_Ung.

### 2.17. Protein-Based Analysis of Ugi Mutants

The construct pBUGI8 [49] and its library mutagenesis products were expressed at a small scale. Pellets from 250 µL aliquots of cell culture were resuspended in 16 µL 1× UDG buffer (NEB) containing lysozyme (50 µg mL^−1^), RNase (40 µg mL^−1^), and 1× Protease Inhibitor Cocktail Tablet solution (Roche). Resuspended pellets were then processed with 3× freeze–thaw cycles at −80 °C (3 min each) and 37 °C (30 s each). Ice cold streptomycin sulphate (Sigma) was added from a 10% (*w*/*v*) stock to a final concentration of 1% (*w*/*v*), with mixing by repeated gentle inversion, and tubes were further incubated on ice for 30 min. Tubes were then centrifuged at 8000× *g* for 2 min at 4 °C; 1.8 µL of the supernatant was extracted and diluted tenfold in UDG buffer supplemented with 100 mM EDTA and 1 unit of *E. coli* UDG. After allowing UDG–lysate interaction at room temperature for 3 min, 1 ng µL^−1^ of thymine-DNA or 2 ng µL^−1^ of uracil-DNA substrate was added to a final volume of 18 µL and incubated at 37 °C for 15 min. Samples were heated at 85 °C for 10 min, then cooled over 20× 30-s steps to 25 °C using the Primus 25 thermocycler (MWG Biotech Inc. High Point, NC, USA). Samples were visualised post 1% agarose gel electrophoresis.

### 2.18. Cellular Survival Selection-Based Analysis of UngIn Functionality

A *dut^−^ ung^−^ E. coli* strain, CJ236, was supplied by Lucigen. This strain has a significant proportion of uracil in the DNA genome; if competent CJ236 cells are transformed with plasmids encoding an *ung* gene, Ung-induced genomic fragmentation results in cell death unless Ung activity is functionally inhibited.

Constructs encoding Ugi, Ung, both Ugi and Ung, or both non-functional Ugi mutant and Ung were developed and used as controls for the cellular survival assay. Control transformations were performed using: (1) vectors carrying Ung-inhibitory genes to produce expected colony counts, (2) vectors carrying both Ung genes and Ung-inhibitory genes to produce fewer than typical colony counts; and a vector carrying uninhibited Ung genes resulting in a lack of observable colonies. This assay was used to test potential UngIn variants and to analyse Ugi library mutagenesis products.

Potential UngIn genes were cloned into constructs carrying an Ung gene to test the Ung inhibition ability of those potential UngIns via the transformation of the plasmid DNA of the verified formed dual constructs into CJ236. Plasmid DNA from any surviving colonies was sequence analysed to reveal UngIn and Ung sequences.

To analyse Ugi mutants, Ugi library mutagenesis of Ugi-Ung-carrying constructs was performed. Circularised products were transformed into NEB^®^ 5-alpha cells. An agar plate containing a well-dispersed lawn of transformed NEB^®^ 5-alpha colonies was treated by spreading 1.5 mL of LB media over the colonies and pipetting the resuspension into a clean tube. The pellet was retained following centrifugation at 8000× *g* for 2 min, and plasmid DNA was isolated using the PureLink™ Quick Plasmid Miniprep Kit (Invitrogen). CJ236 aliquots were transformed with 50 ng of the plasmid DNA. The resulting colonies were grown separately in liquid LB media, and plasmid DNA was analysed via SupremeRun Sanger sequencing services from GATC to obtain data on functional Ugi mutants (Eurofins Genomics).

## 3. Results

### 3.1. A Cellular Survival Assay to Screen for Functional Ung Inhibitors

Two recombinant assays were used to verify the Ung inhibitor (UngIn) function for variants tested in this work: A previously employed biochemical assay based on agarose gel electrophoresis [17] and a newly developed agar plate assay involving the rescue of an *E. coli* mutant cell phenotype that is non-viable in the presence of an active Ung. In the latter, referred to in this work as a cellular survival assay (CS-assay), a *dut^−^/ung^−^* mutant *E. coli* strain CJ236 is used for the transformation of plasmids (Supplementary file—Section S2). Surviving colonies’ plasmid DNA is sequenced to determine the UngIn-encoding sequence.

This assay is employed to ascertain: (1) UngIn functionality in distant homologs of known UngIns, identified from sequence databases; (2) the extent of tolerable mutations at various sequence positions of validated UngIns; (3) potential UngIn activity in new sequences fulfilling certain heuristic signatures typical of UngIns.

### 3.2. Distant Sequence Homologs of p56, SAUGI, and Ugi Are Functional UngIns

In this study, putative UngIn sequences were verified as inhibitors through three different methods. The above two biochemical assays were used: CS-assay in vivo as well as the in vitro agarose gel electrophoresis assay with purified protein. In addition, structural evidence for Ung inhibition was provided by the co-crystallization of putative UngIn proteins with representative Ung enzymes and a structure solution by X-ray crystallography.

**Figure 1:**
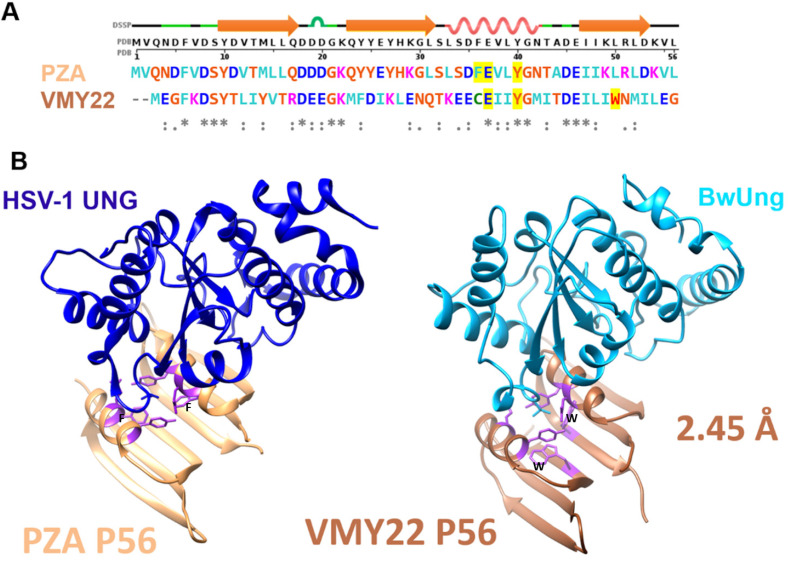
**A comparison of p56 variant structures.** (**A**) Structure-based sequence alignment of PZA p56 and VMY22 p56 with DSSP secondary structure of PZA p56 (PDB: 4L5N) on the top row. (**B**) HSV-1 UNG:p56 (PZA) complex structure (PDB:4L5N) and BwUng:p56 (VMY22) complex structure (new data presented in this study; PDB: 8AIL). The p56 hydrophobic pocket serves to sequester an Ung catalytic residue (a leucine in both HSV-1 UNG and BwUng, shown as sticks on the cartoon in panel (**B**)). The mirror residues comprising the hydrophobic pocket are highlighted yellow in the sequence alignment (panel (**A**)) and are shown in purple in the ribbon representation of both p56 variants (panel (**B**)). A functionally significant phenylalanine residue (labelled F) in the helix of PZA p56 is absent in VMY22 p56; the function of this residue is compensated by a tryptophan residue (labelled W) in the 3rd β-strand of VMY22 p56 subunits. The hydrophobic pocket is thus notably deeper in VMY22 p56.

**Figure 2:**
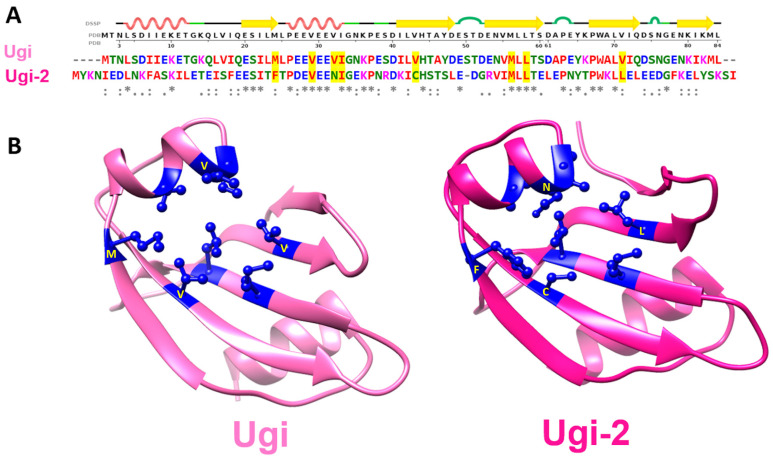
**Ugi variant hydrophobic pockets.** (**A**) Structure-based sequence alignment of Ugi (accession: YP_009664501.1) and Ugi-2 (accession: ALN97938) with DSSP secondary structure of Ugi (PDB: 1UDI) on the top row. (**B**) Ugi structure (PDB:1UDI) and Ugi-2 structure (new data presented in this study; PDB: 8AIM). Both the Ugi and Ugi-2 structures contain a hydrophobic pocket serving to sequester a conserved catalytically critical residue (most frequently a leucine or phenylalanine), employed by Ungs to intercalate DNA via the minor groove and stabilise the pre-catalytic complex. The residues comprising the hydrophobic pocket are highlighted yellow in the sequence alignment (panel (**A**)) and are shown in dark blue in the ribbon representation of both Ugi variants (panel (**B**)). The residue positions are structurally conserved and preserve function; however, the residue types are altered as highlighted yellow in panel (**A**) and labelled yellow in panel (**B**).

**Figure 3:**
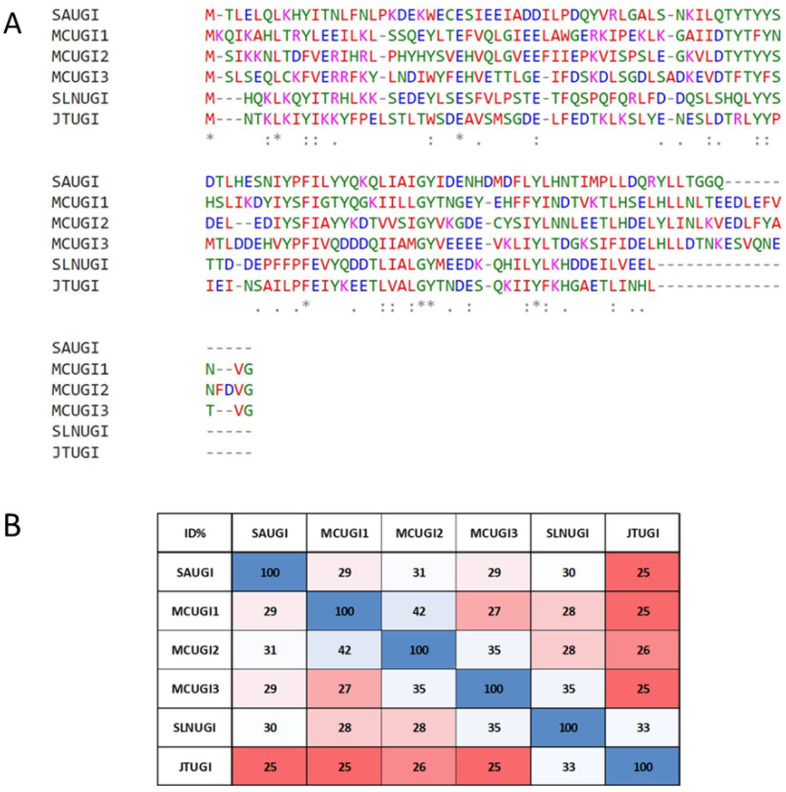
**Sequence plasticity in homologs of SAUGI**. (**A**) Clustal-format-structure-based multiple sequence alignment of SAUGI and the identified weakly homologous sequences (SYUGI, accession: WP_052256111.1; JMUGI, accession: WP_185124884.1; MBUGI, accession: WP_165958605.1; MCUGI1, accession: WP_101156358.1; and MCUGI2, accession: WP_101143899.1). Multiple sequence alignment was performed online using MAFFT at the EMBL-EBI webserver. Among the 112 amino acid residues in SAUGI sequence, only 6 residues, excluding the start codon translated methionine, remain identical in all six variants. (**B**) Sequence identity matrix between SAUGI variants. This matrix highlights that no pairs among these six distant homologs share > 42% identity. Sequence identity amongst these weakly homologous SAUGI variants can be as low as 25%.

(1) Using PZA p56 (Accession: NP_040721.1) as a template sequence with the previously identified PROSITE pattern E-X(2)-Y-X(0,2) G [16] returned 19 sequences from a PHI-BLAST homology search (Supplementary Figure S1). Of these, the most distantly related to structurally characterised p56 proteins is a sequence encoded by Bacillus phage VMY22 (“VMY22_4”; Gene ID: 26625151), with a sequence identity of 23% to Bacillus phage PZA p56. The VMY22 open reading frame sequence was synthetically optimised for *E. coli* and recombinantly expressed. The protein was biochemically (Supplementary Figures S2–S4) validated to act as an UngIn and confirmed as a structural homolog of previously characterised p56 proteins (Figure 1).

(2) Using the PBS1 Ugi sequence (accession: YP_009664501.1) as a query for a PSI-BLAST search, four output homologous sequences were recovered, each annotated as uracil-DNA glycosylase inhibitor (Supplementary Figure S5). The weakest homolog is encoded by Bacillus phage vB_BpuM-BpSp (locus_tag = “Bp8pS_259”; accession number ALN97938) and 32% of its identity is composed of Ugi (as encoded by Bacillus phages AR9, PBS1 and PBS2). This sequence (referred to henceforth as Ugi-2) was biochemically (Supplementary Figures S2–S4) and biochemically/structurally (Figure 2) validated to act as an UngIn.

(3) Using the prototypical SAUGI sequence (UniProtKB Q936H5_STAAU) as a query for a PSI-BLAST search (limiting the search to family *Staphylococcaceae*) returned 1024 sequences. Searches were limited to *Staphylococcaceae* to improve sensitivity—this returned two extra hit sequences which were not found when searching against all reference genomes. Applying thresholds of >80% query cover and <35% percent identity to the query, 15 non-redundant sequences were selected from *Macrococcus* sp. (13 sequences, in three discrete sequence groups; Supplementary Figure S6), *Salinicoccus* sp. YB14-2 (1 sequence), and *Jeotgalicoccus meleagridis* (1 sequence). Multiple sequence alignment was performed online using MAFFT on the EMBL-EBI webserver [27,51]. These output sequences were designated MCUGI1, MCUGI2, MBUGI, SYUGI, and JMUGI; all are significantly diverse with respect to SAUGI and each other, with pairwise identities ranging from 25% to 42% (Figure 3).

**Figure 4:**
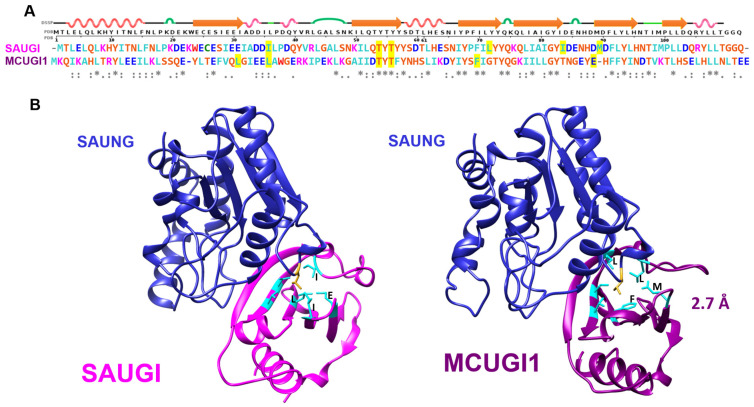
**SAUGI and MCUGI1 hydrophobic pockets.** (**A**) Structure-based sequence alignment of SAUGI (UniProtKB Q936H5_STAAU) and MCUGI1 (accession: WP_101156358.1) with DSSP secondary structure of SAUGI (PDB: 3WDG) on the top row. (**B**) SAUNG:SAUGI complex structure (PDB:3WDG) and SAUNG:MCUGI1 complex structure (new data presented in this study; PDB: 8AIN). SAUGI and MCUGI1 hydrophobic pockets serve to sequester a conserved catalytically critical residue (a leucine in SAUNG, coloured red-orange in panel (**B**)) employed by Ungs to intercalate DNA via the minor groove and stabilise the pre-catalytic complex. The residues comprising the hydrophobic pocket are highlighted yellow in the sequence alignment (panel (**A**)) and are shown in cyan in the ribbon representation of both SAUGI and MCUGI1 (panel (**B**)). UngIn function is preserved, despite the fact that residue positions are neither sequence- nor structure-conserved (as labelled in panel (**B**)).

Variant SAUGI gene loci were found in novel permutations of the SCC*mec* cassette (Supplementary Figure S7). The sequences MCUGI1, MCUGI2, MBUGI, SYUGI, and JMUGI were biochemically validated to act as UngIns (Supplementary Figures S2–S4); the sequence MCUGI1 was also structurally validated to act as an UngIn by co-crystallisation with SAUNG (Figure 4).

**Figure 5:**
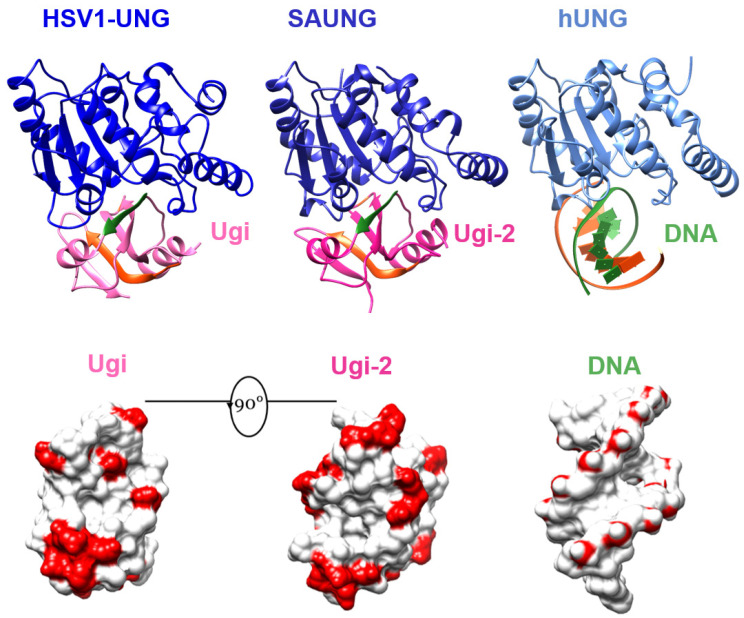
**DNA mimicry by Ugi variants. Top row left to right**: The structure of HSV1-UNG with PBS1 Ugi (PDB: 1UDI), the structure of SAUNG in complex with Ugi-2 as determined in this study, and the structure of hUNG with dsDNA (PDB: 1SSP). DNA backbone 1st strand (coloured green) is mimicked by the 1st β-strands (coloured green) of both Ugi variants’ β-sheet. DNA backbone second strand (coloured orange) can also be traced within both Ugi variants coloured orange in both Ugi variants. **Bottom row**: the Ung-binding interfaces of PBS1 Ugi, Ugi-2, and dsDNA. Bottom row depictions were generated by rotating top row view by 90° degrees, removing Ung chains for clarity, and then showing the surfaces of Ung-bound molecules. The red colour in bottom row corresponds to the acidic residues of Ugi variants and the phosphate groups of dsDNA. A similar pattern of negative charge distribution can be observed in the three different molecules.

In summary, recombinant analysis of the VMY22 p56, Ugi-2, and MCUGI1 gene products revealed that each could functionally inhibit Ung when assayed in vitro, and each supported *E. coli* CJ236 colony formation in the cellular survival assay presented in this study. Furthermore, crystal structures were determined for each variant in complex with Ung and revealed fold conservation with PZA p56, SAUGI, and Ugi. Ung contacts related to UngIn function were conserved, with global RMSD values in chain overlays as follows: VMY22 p56 vs. PZA p56 = 0.883 Å (Figure 1); MCUG1 vs. SAUGI = 1.005 Å (Figure 4); Ugi-2 vs. Ugi = 1.343 Å (Figure 5). Nevertheless, critically important residues at the Ung-binding interface and in the hydrophobic inhibitor pocket show sequence plasticity relative to reference sequences (Bacillus phage PZA p56, SAUGI, and Ugi), and it is observed that residue side chains from alternative sequence positions can spatially compensate to conserve function when the local mutation is functionally unfit.

### 3.3. Plasticity of Canonical Sequence Motifs in p56 Variants

PZA p56 and VMY22 p56 variants show structural conservation (RMSD = 0.883 Å) despite low sequence identity (24%). The primary difference of note is a deeper inhibitory hydrophobic pocket in the VMY22 p56 variant, due to tryptophan in the 3rd β-strand functionally substituting a helix-located side chain that is a phenylanaline in PZA p56. In VMY22 p56, that phenylalanine is a cysteine and does not form part of the hydrophobic pocket. Other sequence changes do not contribute to significant observed structural differences.

### 3.4. Plasticity of Canonical Sequence Motifs in Ugi Variants

Ugi and Ugi-2 variants show structural conservation (RMSD = 1.343 Å) despite low sequence identity (32%). Although not identical, the negative charge distribution on the surface of each Ugi variant effectively mimics the negative charge distribution of phosphate groups on Ung-bound DNA; in addition, the grooves of DNA are mimicked by depressions on the surface of each Ugi variant (Figure 5). Ugi-2 has 21 acidic residues in its primary sequence, while PBS1 Ugi has 18 acidic residues. There are only eight positions of conserved negatively charged residues between the two Ugi sequence variants, of which, five are within the Ung-binding β-strand 1 and α-helix 2 in a 12aa span (Supplementary Figure S8). A PROSITE pattern of E-X(6)-[ED]-[ED]-X-[ED]-[ED] may thus be defined for these Ugi variants.

The Ung-sequestering hydrophobic pocket of PBS1 Ugi is formed by eight residues (Figure 2), of which, only four are identical in Ugi-2. This provides important data on tolerated variations, including the essential residues that perform Ung inhibition.

Sequence diversity is not limited to the loops and other flexible secondary structures. It can be observed in some core-forming secondary structures such as the second β-strand (_41_ILVHTAYD48 in PBS1 Ugi, and _45_KICHSTSL_52_ in Ugi-2) in which only the histidine residue is common to these Ugi variants (Supplementary Figure S8). Within the remaining residues that form this β-strand, four out of seven are non-conservative mutations (I41 Ugi/K45 Ugi-2; V43 Ugi/C47 Ugi-2; Y47 Ugi/S51 Ugi-2; D48 Ugi/L52 Ugi-2).

### 3.5. Sequence Plasticity in Canonical Sequence Motifs in SAUGI

The structural conservation between SAUGI and MCUGI1 variants is observed to be close with RMSD = 1.005 Å at low levels of sequence identity (29%). SAUGI contains 17 acidic residues, while MCUGI1 has 18 acidic residues. Just five such positions of negative charge are common to both sequences, of which, three are located within a 10-residue span in the Ung-binding β-strand 1 and α-helix 2. The PROSITE pattern of these acidic residues, E-X(6)-[ED]-[ED], is also common to Ugi and Ugi-2. Significant sequence plasticity is also observed in the Ung-binding β-strand. Of eight residues comprising the Ung binding β-strand, _24_ECESIEEI_31_ in SAUGI and _24_LTEFVQLG_31_ in MCUGI1, only a single glutamic acid residue is common to these variants and five residues comprise non-conservative variation (Supplementary Figure S9).

The Ung-sequestering hydrophobic pocket of SAUGI is formed by six residues (Figure 4), of which, only three are identical in MCUGI1. The other three residues forming the hydrophobic pocket in MCUGI1 do not align either at the sequence or structure level with those forming the hydrophobic pocket in the prototypical SAUGI, although residue side chains do occupy the same position in space. These sorts of variations between hydrophobic pockets could nevertheless prove informative for the exploration of sequence space for SAUGI variants.

**Figure 6:**
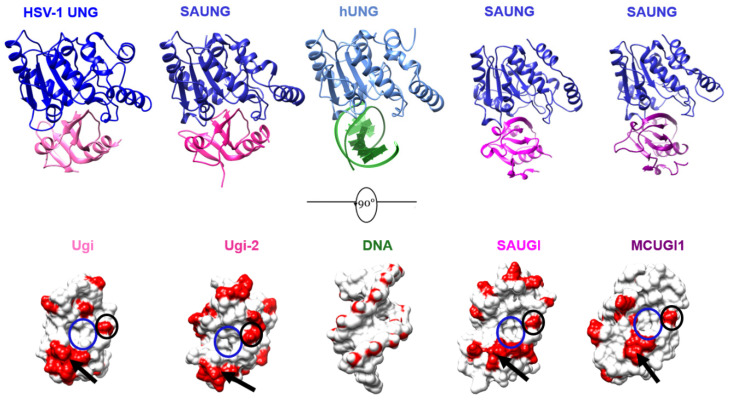
**Structural properties of Ugi/SAUGI variants. Top row left to right**: The structure of HSV1-UNG:UGI (PBS1) (PDB: 1UDI); the structure of SAUNG:Ugi-2 presented in this study (PDB: 8AIM); the structure of hUNG with dsDNA (PDB: 1SSP); the structure of SAUNG:SAUGI (PDB: 3WDG); the structure of SAUNG:MCUGI1 presented in this study (PDB: 8AIN). **Bottom row left to right**: the Ung-binding interfaces of Ugi, Ugi-2, dsDNA, SAUGI, and MCUGI1. **Bottom row** depictions were generated by rotating each top row view by 90° degrees, removing Ung chains for clarity, and then showing the Ung-bound surfaces of molecules. The red colour in the bottom row corresponds to the acidic residues of Ugi/SAUGI variants and the phosphate groups of dsDNA. An identical glutamic acid residue in the 1st β-strand of Ugi/SAUGI variants (black circle), an acidic motif (black arrow), and a hydrophobic pocket (blue circle) are structurally conserved properties among all 4 Ugi/SAUGI variants presented in this figure.

The surface negative charge distribution of MCUGI1 is somewhat different to that in Ugi, Ugi-2, and SAUGI. However, common properties among all these four Ugi/SAUGI variants include an invariant glutamic acid residue, a conserved acidic motif, and a spatially conserved hydrophobic pocket (Figure 6).

### 3.6. Random Mutagenesis of Ugi to Assess Tolerance of Viable Plasticity

Since Ugi and SAUGI share a common fold from heterogeneous sequences, a structure-based sequence alignment approach was used to design random substitutions at defined positions in the sequence. A library mutagenesis approach was used to sample residue tolerance at these critical positions. The deposited crystal structures of HSV-1 UNG complexes with PBS1 Ugi [43] (PDB: 1UDI) and SAUGI [42] (PDB: 5AYS) were used as templates for the alignment of these UngIns (Supplementary Figure S10).

A motif comprised of three amino acids (ESI) was found to occur in common in both Ugi and SAUGI. This motif is found in the first β-strand of these UngIns, which docks in the Ung-DNA binding cleft. In the SAUGI sequence family, it is observed that the ESI motif tolerates mutations at positions 2 (Ser) and 3 (Ile), according to the pattern E-[APST]-[FILMV]. The first β-strand of Ugi and the ESI motif in Ugi were separately sampled via library mutagenesis to discover tolerated variations.

Libraries were designed to randomly mutate the residues comprising the Ung-binding β-strand of Ugi (library L1), to shuffle the ESI motif according to observed variation in SAUGI sequence data (library L2), and to randomly mutate the ESI motif (library L3). The transformation of library L1 into the *E. coli* strain CJ236 (i.e., CS-assay) resulted in no surviving colonies being observed (i.e., no synthetic Ugi sequences with a novel first β-strand sequence conserving UngIn function).

A transformation of library L1 into the *E. coli* strain NEB 5α, followed by small-scale expression and soluble fraction visual analysis via Coomassie-stained SDS-PAGE, identified two colonies (designated as clones U10 and U12) that were found to encode soluble recombinant protein. Sanger sequencing of U10 and U12 revealed Ugi encoding sequences with an altered sequence in the first β-strand. The U10 and U12 proteins did not function as UngIns in the CS-assay, and there was no detectable UngIn function when U10 and U12 proteins were purified and assayed in vitro. These variants were therefore used as parental DNA for CS-assay construct manufacture to remove the risk of false positive colonies.

The CS-assay for libraries L2 and L3 yielded a total of 16 unique nucleotide sequences encoding 12 novel Ugi variants. One variant encoded the prototypical Ugi amino acid sequence, differing only in its nucleotide sequence. Importantly, the remaining 15 synthetic sequences showed 11 novel motif sequences substituting for the Ugi-type ESI motif without loss of UngIn functionality (Table 2).

### 3.7. Using Insights from Synthetic Ugi Variants in Bioinformatics Sequence Searches

Insights from tolerated mutations at the ESI motif of Ugi/SAUGI UngIns were utilised to search for novel UngIn sequences. Each synthetic Ugi variant, discovered from library mutagenesis, was used as a search sequence along with a PROSITE pattern representing the Ung-binding β-strand motif. A PHI-BLAST search in the *Myoviridae* family with an EAM motif in place of ESI returned no Ugi sequences but did return a sequence encoded by the uracil-DNA *Staphylococcus* phage MarsHill, with 31% identity of the synthetic EAM Ugi variant (Supplementary Figure S11). This hit could not be detected using wild-type Ugi variants in any PSI-BLAST or PHI-BLAST search.

Upon CS-assay analysis, no surviving colonies of *E. coli* CJ236 were recovered from any of these recombinant sequences, which suggests that these sequences do not encode UngIns.

### 3.8. Using Insights from Synthetic Ugi Variants to Develop Heuristic Search Parameters for Genomic UngIn Discovery

A conserved motif in the β-strand of Ugi and SAUGI UngIns that docks the Ung DNA binding cleft is defined as ESI in prototypic variants. This ESI motif was redefined via a curated MSA involving all available SAUGI-type sequences (sequences available in GitHub repo, see Section 2) created using structure-based alignment with verified Ugi and SAUGI variants, assuming that close sequence homologs of any validated variants could also be functional UngIns. Thus, the ESI motif can be expressed as E-[ASVFHTNI]-[LVIFMT]. Adding insights from synthetic library Ugi variants, the third position of the ESI motif may be redefined as [LVIFMTPWC]. Such insights were used as the foundation of a heuristic to define Ugi/SAUGI type UngIns in sequence space. The analysis of hydrophobicity, acidity, local molecular mass bounds, glycine/proline content, and glutamic acid/aspartic acid content within Ugi/SAUGI sequences defined specific ranges for each of the analysed attributes (Supplementary Figure S12). The set of heuristic attributes was utilised to build a series of filters for the discovery of genomic UngIn sequence signatures.

### 3.9. Design and Optimisation of the Ugi/SAUGI Heuristics-Based Filter Pipeline

Genomic nucleic acid sequences were translated in all six reading frames, and the resulting sequences were triaged via a pipeline consisting of: a protein length filter, an acidity filter, a hydrophobicity filter, a characteristic glycine/proline residue content filter, and an acidic-to-basic residue ratio filter (Supplementary Table S3). A subsequent filter was finally applied to remove sequences without an ESI motif. In parallel triage pipelines, ESI-less was modified to scan for the absence of either a strict natural E-[ASVFHTNI]-[LVIFMT], lenient E-[X]-[LVIFMT], or natural + synthetic at position 3 [LVIFMTPWC], whether strict or lenient in the first two positions of the motif. Filters and parameters were tested in development using uracil-DNA phage genome sequences encoding verified Ugi-type UngIn sequences so that heuristic filters were optimised for the discovery of Ugi-type UngIns (Supplementary Figure S13). Heuristics filters were tuned such that all known Ugi-type sequences were returned with as few false positives as possible. More lenient filters were applied when filtering single genomes, but when examining large files consisting of translations from thousands of genomes, stricter parameters were utilised to return a manageable number of heuristic matches for recombinant analysis (Supplementary Tables S3 and S4).

### 3.10. Ugi-Heuristic Matches from Yersinia Phage PhiR1-37

**Figure 7:**
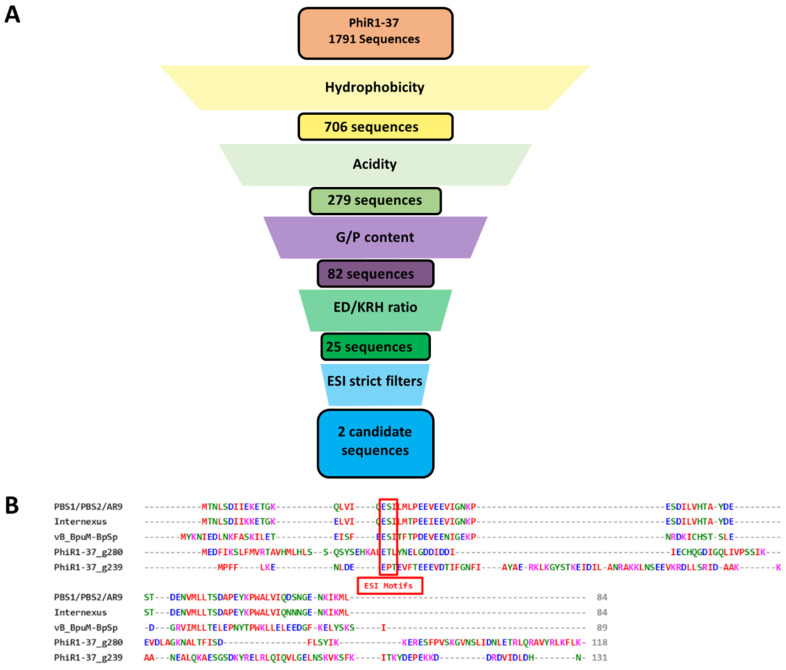
**Candidate Ugi-heuristic matches from *Yersinia* phage PhiR1-37**. (**A**) The filter pipeline returned two candidate sequences out of 1791 possible protein-coding sequences. (**B**) MSA including PhiR1-37 heuristic triaged sequences, aligned against known Ugi sequences from phages PBS1/PBS2/AR9, Internexus, and vB_BpuM-BpSp. The two candidate heuristic matches have ESI motifs (boxed in red in the MSA) that satisfy all heuristic parameters.

The filter pipeline was used to search translated polypeptide sequences from the uracil-DNA genome of *Yersinia* phage PhiR1-37 as an input. Filters returned two sequences matching the heuristic demands (Figure 7). The sequences each contain an ESI motif as expected. Recombinant analysis of synthetic open reading frames encoding the triaged sequences via the CS-assay did not return any colonies and thus does not indicate UngIn function in vivo. In vitro use in a visual Ung assay revealed these proteins apparently do not act as UngIns.

### 3.11. Ugi-Heuristic Matches from Myoviridae

All *Myoviridae* family genomes were used as input for the filter pipeline with the strictest parameters, including strict ESI filtering (Supplementary Table S4). Genomes were also binned by %GC content, since it is observed that all verified Ugi variants are found in genomes with GC content between 25 and 30%. Phage genomes encoding UngIns were therefore assumed to consist of lower %GC content relative to other phages, which is a focus of the discussion in this manuscript. In support of this assumption, %GC bins from 25–35% (Supplementary Figure S14) returned the most heuristic output sequences. Stricter ESI-motif filtering was applied to limit the number of outputs for each %GC bin.

Heuristic pipeline searches within *Myoviridae* genomes did not return any output that could putatively be assigned as an UngIn. Other than those encoding Ugi sequences, no uracil-DNA genome (i.e., *Yersinia* phage PhiR1-37 [21], *Staphylococcus* phages Machias, MarsHill, Madawaska [22], and S6 [52], and *Listeria* phage LPJP1 [53]) outputs were obtained. Thus, the filter pipeline was serially modified to be more lenient by removing filters ad hoc in turn, and two output sequences were returned when the glycine/proline residue count filter was inactivated; these sequences originated, respectively, from the uracil-DNA *Staphylococcus* phage S6 and from *Listeria* phage LPJP1 (Supplementary Figure S15).

### 3.12. UngIn Assay Testing of Proteins Conserved Exclusively in Uracil-DNA Phages

Reference genome sequences (GenBank) for bacteriophages reported to employ uracil-DNA genomes (*Staphylococcus* phages MarsHill, Madawaska, and Machias, *Listeria* phage LPJP1) were analysed via a TBLASTN search with the PhiR1-37 genome as a query to define mutually conserved proteins of unknown function. Seven such proteins were found to be common to these phages; two of these seven proteins (genes g278 and g282 of PhiR1-37) are homologs of each other and are also found in the other named uracil-DNA genomes as a pair of homologous proteins. A PSI-BLAST search was performed for each of these seven proteins to ascertain whether their genomic presence is exclusive to uracil-DNA phages. It was found that six of the proteins are indeed limited to uracil-DNA phages (Table 3), including phages known to encode Ugi, as well as two closely related *staphylococcus* phages, respectively, PALS2 [54] and vB_StaM_SA1 [55], exhibiting high degrees of genome similarity to uracil-DNA phages.

Uracil-DNA *Myoviridae* phages are known to encode a non-canonical RNA polymerase that recognises uracil-containing promoters [19]. Performing a PSI-BLAST search using this RNA polymerase as an input (encoded by AR9 uracil-DNA phage; accession: YP_009283131.1) returns homologous sequences from known uracil-DNA phages in addition to homolog sequences from PALS2 and vB_StaM_SA1 phages, further implicating them as putative uracil-DNA phages.

The six identified uracil-DNA phage-exclusive genes were synthesised for optimised recombinant *E. coli* expression and cloned into pSDM4_U12_Ung via OE-PCR. However, none of these supported Ung inhibitory activity via the CS-assay (see Section 4).

### 3.13. Analysis of p56-like Sequences to Assess UngIn Function

A PSI-BLAST search against the non-redundant database using the VMY22 p56 as a query returned no new p56 sequences. However, limiting the search to the *Salasmaviridae* family increased the sensitivity to identify additional homologous sequences after four iterations in the closely related genomes of *Northropvirinae* viruses, including *Bacillus* phages DK1, DK2, DK3 [56], and vB_BthP-Goe4 [57] (Supplementary Figure S1). These phages exhibit significant genomic and proteomic similarity to p56-encoding phages. The PSI-BLAST output sequences are longer than p56, thus partially aligning to conserved portions of known p56 sequences, which is a known feature of the validated UngIn encoded by *Bacillus* phage GA-1 (Supplementary Figure S1) [58].

Fragments of these PSI-BLAST hits showing homology to VMY22 p56 were used in recombinant assays but failed to show UngIn activity (Supplementary Figure S4). However, high-confidence AlphaFold2-predicted structures of these homologs showed significant similarity to the p56 protein fold (Supplementary Figure S16).

Variation between p56 and *Northropvirinae* homologs appears in motifs crucial to Ung inhibition, particularly the p56 E-X-X-Y motif where a tyrosine that is crucial for dimerisation and hydrophobic pocket formation is mutated to isoleucine. Another motif, not apparently involved in supporting UngIn function, F-X-D-S-Y, is conserved in all p56 UngIns, although not in the *Northropvirinae* homologs. *Northropvirinae* encode a dUTPase, which the *Salasmaviridae* lack. This may be significant and is considered in the discussion.

## 4. Discussion

In this study, we primarily addressed the question of why Ung-inhibitor (UngIn)-encoding sequences appear to be absent in the genomes of uracil-DNA phages.

Viruses and transposable genetic elements can encode proteins that modulate the activity of uracil-DNA glycosylase (UDG). To date, the Ung-type UDGs are the only known targets of such specialised UDG-inhibiting proteins. UngIns are described as DNA-mimicking proteins that specifically sequester the Ung minor groove binding loop via a hydrophobic trap and disable Ung, which is the primary conduit for uracil-DNA repair. UngIns appear to have independently arisen in viruses from diverse protein folds on at least three occasions, and their observed diversity at the primary structure level would suggest that the prevention of uracil-DNA repair invokes significant evolutionary pressure. This in effect obscures UngIn signatures at the genomic level and creates a genomic palimpsest.

We investigated sequence plasticity relating to the Ugi-, SAUGI-, and p56-type UngIns via bioinformatics, structural biology, structure prediction, and library mutagenesis, as well as the computational heuristic triage of genomes and validation via recombinant expression in *E. coli* and assay for UngIn function.

Table 4 summarises the primary results of this survey. Of interest are some discovered SAUGI variants, residing in unique loci in the SCC*mec* cassette. Genomic context could be useful in annotating putative UngIns, even when sequence homology is very low. SAUGI is a conserved gene in the transposable genomic pathogenicity island SCC*mec* of methicillin-resistant *Staphylococcus aureus* (MRSA) strains, specifically on the cassette chromosome recombinase (ccr) complex [59]. Genomic mapping of regions flanking the discovered SAUGI variants confirmed that they reside in the ccr complex of SCC*mec*. Unusually, SYUGI and JMUGI were found to be located adjacent to the DNA repair protein radC (Supplementary Figure S6).

We have been able to verify that distant sequence homologs of p56, SAUGI, and Ugi are UngIns. We have not, however, been able to validate any new gene locus as encoding UngIn function in any phage genome accession including uracil-DNA phages. Below are discussed some possible reasons for these results, which we would assume are due to the precarious evolutionary strategy of early-phase Ung inhibition in virus replication.

It is possible that negative results observed in biochemical assays are false negatives owing to the choice of Ung used. The CS-assay in this work screens for UngIn activity against HSV-1 Ung; however, it is conceivable that putative UngIn sequences may display activity against Ung enzymes from different species. This potential limitation notwithstanding, it should be noted that all known UngIns do indeed bind HSV-1 Ung both in vitro and in the CS-assay.

From an evolutionary perspective, uracil-DNA protection strategies may cause the genomic G+C content in viral progeny to drop, as cytosine deamination events go unrepaired and the rate of C:G to T:A transitions goes up. Phage genomes generally show a lower G+C content than their host [60]. This disparity in G+C content would appear to drive codon usage, which in turn exerts pressure on proteomic architecture [61,62]. A mutator phenotype that drives these transition mutations (such as conditions prevailing under an UngIn gene product) would sample mutation landscapes even if overall fitness is compromised [63]. Under extreme pressure towards mutation, which may exist in lineages that always suppress uracil-DNA repair, a neutrality paradox will manifest, which biases towards non-conservative mutation landscapes [64].

Nevertheless, there is a theoretical limit to how much attrition is possible because the universal genetic code still requires cytosine or guanine bases to encode all natural amino acids. Furthermore, small amino acids such as glycine, proline, and alanine, are encoded by GC-rich codons, which are under-represented in low-GC genomes and have the lowest α-helical and β-sheet inclusion propensities in protein structures [65,66]. These residues tend to break such secondary structures, yet glycine and proline in particular help to increase the conformational stability of proteins when they are abundant in β-turns [67]. The under-representation of such amino acids in low-GC-content genomes is therefore unaffordable and would also be expected to act as a limit on genetic fitness.

However, since phage GC content is generally related to that of the host cell, an opposing evolutionary force must be in operation [68]. In the case of uracil-DNA phages, the loss of an UngIn phenotype would be lethal, notwithstanding that the Ung inhibitor creates an extreme selection landscape driving sequence variation, with stochastic loss and gain of encoded functions [62,64,69]. It is thus likely, given the extreme evolutionary pressures associated with Ung inhibition, that UngIn functions are unfavourable to viruses that are not sensitive to uracil arising spontaneously in their genomic DNA.

Viruses that make use of UngIn functionality would likely require backup or alternative protective strategies in case this inhibition function is lost by mutation. This could include the encoding of redundant UngIn proteins at more than one gene locus, ensuring protection in case any individual protein loses inhibitory function. It might also rely on other types of moderating factors. Sequence plasticity in any UngIn lineage would probably depend upon environmental factors influencing the type of mutations that arise [60,62]. The notion of redundancy in UngIn functionality might also explain how several protein folds came to support the Ung inhibition. In jumbophages, only Ugi-type UngIns are known to be encoded at a single locus and exclusively by *Bacillus* infecting jumbophages [70]. There is no evidence of genes encoding variants of p56 or Vpr UngIns in these phages. However, uracil-DNA phages infecting other species appear to lack an encoding gene for any known UngIn type and therefore present a paradox, because the genome biochemistry would suggest that an UngIn is required.

Uracil-DNA phages lacking obvious candidates for an UngIn encoding gene include *Yersinia* phage PhiR1-37 [21], *Staphylococcus* phages MarsHill, Madawaska, Machias [22] and S6 [52], and *Listeria* phage LPJP1 [53], as well as two phages which we propose display characteristics associated with uracil-DNA phages: PALS2 [54], and vB_StaM_SA1 [55]. Could these phages encode one or more novel UngIn functions? Any such novel UngIn types could also conceivably exist as backups in the *Bacillus* phages encoding Ugi. SAUGI variants are considered to have a phage origin and are structurally related to yet divergent from *Bacillus* phage Ugi variants, although SAUGI-type sequences are not present in any phages considered here.

A solution to the enigma may be presented by factors other than UngIns that could promote alternative routes to survival. For example, *Salasmaviridae* phages Phi29 and PZA and the *Tatarstanvirinae* phage GA-1 are known to encode p56 UngIns [15,17,58], despite not utilising uracil-DNA. This may act to protect single-strand intermediates formed during the protein-primed DNA replication of these phages [15]. However, despite the presence of p56 sequence homologs in the closely related genomes of *Northropvirinae* viruses, including *Bacillus* phages DK1, DK2, DK3 [56], vB_BthP-Goe4 [57], and vB_BceP-DLc1 [71], the recombinant assays performed in this study suggest these *Northropvirinae* genes do not encode an UngIn. Variations in motifs crucial to UngIn function likely explain this phenotype. In contrast to *Salasmaviridae* genomes, the *Northropvirinae* genomes are found to encode a dUTPase, which would prevent the replicative misincorporation of uracil in place of thymine. If the phage dUTPase is lost, selection for mutations to the p56 homolog gene that convey p56-type UngIn function would increase replication fitness via Ung inhibition.

In this work, we did not discover any UngIn functionality via heuristic signature matching based upon verified UngIn properties; thus, we focused instead on gene loci common only to uracil-DNA phages. Of those genes (Table 3) none expressed proteins functioning recombinantly as UngIns. There are two principal possibilities: (1) There is a novel type of UngIn family, which may be a monomer or homopolymer, or even require heteropolymeric assembly via the products of multiple gene loci. (2) UngIns are not the only route to survival for uracil-DNA phages. For example, perhaps the second endonucleolytic step in BER can be a valid target for inhibition. This latter point comes with the caveat that multiple proximal abasic sites in DNA due to Ung activity are considered unstable.

In summary, the CS-assay provides a rapid means of testing for UngIn functionality of recombinant samples. It is clear, however, that the structure- and function-informed heuristic sequence search approach presented here is limited in that novel types of UngIn fold cannot be searched. It is therefore suggested that a suitable alternative may be provided via recent developments in structure prediction methods [46,48,72,73]. New families of UngIn, or other Ung-modulating factors, could be revealed by proteome-scale structure prediction for uracil-DNA phages, combined with multimer prediction methods to allow the visualization of putative interfaces with Ung. With experimental verification via CS-assay, such an approach may provide the route to expanding our knowledge of adaptation to support the evolutionary twilight zone created by UngIn deployment in support of virus replication.

## Figures and Tables

**Table 1:** X-ray diffraction data collection and refinement statistics.

	Ugi-2:SAUNG	MCUGI1:SAUNG	VMY22p56:BwUng
PDB accession code	8AIM	8AIN	8AIL
**Data collection**			
Wavelength (Å)	0.9795	0.919763	0.976250
Space group	C 2 2 21	P 63 2 2	P 1 21 1
Unit cell a, b, c (Å)	137.65, 142.82, 82.76	91.52, 91.52, 158.62	85.327, 97.495, 100.555
α, β, γ (°)	90.00, 90.00, 90.00	90.00, 90.00, 120.00	90.000, 111.362, 90.000
Resolution (Å)	52.92–2.60 (2.72–2.60)	45.80–2.70 (2.83–2.70)	48.87–2.45 (2.52–2.45)
Unique reflections	25,462 (3052)	11,391 (1470)	56,337 (4587)
Redundancy	1.9 (1.9)	11.9 (12.6)	4.4 (4.3)
Completeness (%)	100 (100)	100 (100)	99.8 (99.7)
I/σ(I)	4.1 (1.64)	9.8 (2.12)	9.8 (2.53)
*R*_merge_	0.121 (0.588)	0.251 (1.682)	0.109 (0.548)
**Refinement**			
*R*_work_ (%)	0.202	0.208	0.204
*R*_free_ (%)	0.246	0.256	0.237
Bond RMSD (Å)	0.007	0.0078	0.0074
Angle RMSD (°)	1.469	1.586	1.413
Mean			
B value/no of atom	46/9750	49/5286	36/21,921
Ramachandran plot (%)			
Most favoured (%)	95.29	92.06	97.22
Allowed (%)	4.71	7.94	2.78
Outliers (%)	0.00	0.00	0.00

**Table 2:** Library mutagenesis targeting the Ung-binding 1st β-strand of Ugi.

**Sequences of 1st β-strand, and library diversity** Ugi: **QESILML** Library 1: **XXXXXXX** Library 2: **Q[ED][APST][FILMV]LML** Library 3: **Q[ED]XXLML**
**Synthetic Ugi sequences generated from mutagenesis libraries** Library1: Soluble mutants lacking UngIn function: U10 **PTRSIVK**, U12 **KSNKSLP** Library2: **QESILML, QEAMLML** Library3: **QEALLML, QESTLML, QESVLML, QETCLML,** **QESWLML, QEAPLML, QETVLML, QEVTLML,** **QETMLML, QETILML**

**Table 3:** Hypothetical proteins exclusively encoded by uracil-DNA phages.

PhiR1-37 Encoded Gene (Length aa, pI)	Sequence Accession	Encoding Phage	ID %	Sequence Length (aa)
g207 (863, 4.3)	YP_004934441.1	phiR1-37	100%	863
	YP_009283116.1	AR9	30%	904
	QXN70134.1	vB_BspM_Internexus	30%	904
	YP_009664305.1	PBS1	30%	904
	ALN97947.1	vB_BpuM-BpSp	30%	903
	QQO92841.1	Madawaska	28%	995
	QQM14726.1	MarsHill	28%	995
	QDJ97626.1	PALS_2	28%	995
	QQO92559.1	Machias	27%	996
	QPI17170.1	vB_StaM_SA1	27%	995
	QXN67971.1	LPJP1	27%	995
g278/g282 (295, 5.0)/(225, 4.8)	YP_004934512.1	phiR1-37	100%	295
	QXN67837.1	LPJP1	45%	174
	YP_009283146.1	AR9	44%	234
	YP_009664335.1	PBS1	44%	234
	QXN70164.1	vB_BspM_Internexus	43%	234
	ALN97995.1	vB_BpuM-BpSp	41%	214
	QQO92450.1	Machias	41%	211
	QXN67771.1	LPJP1	40%	249
	ALN97977.1	vB_BpuM-BpSp	39%	240
	QPI17143.1	vB_StaM_SA1	39%	227
	QDJ97768.1	PALS_2	39%	214
	QQM14590.1	MarsHill	39%	214
	QQO92712.1	Madawaska	39%	214
	QPI17045.1	vB_StaM_SA1	39%	210
	QQO92553.1	Machias	37%	230
	QDJ97877.1	MarsHill	37%	227
	QDJ97877.1	PALS_2	37%	227
	QQO92814.1	Madawaska	37%	227
	YP_009283165.1	AR9	37%	221
	YP_009664352.1	PBS1	37%	221
	QXN70182.1	vB_BspM_Internexus	36%	230
	YP_004934516.1	phiR1-37	35%	225
g234 (846, 4.9)	YP_004934468.1	phiR1-37	100%	864
	QQO92451.1	Machias	28%	848
	QPI17046.1	vB_StaM_SA1	28%	847
	QXN67836.1	LPJP1	28%	823
	YP_009664354.1	PBS1	28%	738
	YP_009283164.1	AR9	28%	738
	QXN70181.1	vB_BspM_Internexus	28%	738
	QQM14591.1	MarsHill	27%	847
	QQO92713.1	Madawaska	27%	847
	QDJ97769.1	PALS_2	27%	847
	ALN97996.1	vB_BpuM-BpSp	27%	734
g244 (290, 9.6)	YP_004934478.1	phiR1-37	100%	290
	QPI17158.1	vB_StaM_SA1	35%	254
	QXN67980.1	LPJP1	33%	173
	QDJ97892.1	PALS_2	31%	227
	YP_009283131.1	AR9	30%	274
	YP_009664320.1	PBS1	30%	274
	QXN70149.1	vB_BspM_Internexus	29%	269
	QQM14714.1	MarsHill	29%	257
	QQO92829.1	Madawaska	29%	257
	ALN97961.1	vB_BpuM-BpSp	29%	254
	QQO92570.1	Machias	28%	255
g196 (850, 7.0)	YP_004934430.1	phiR1-37	100%	850
	ALN97953.1	vB_BpuM-BpSp	26%	408
	QXN70140.1	vB_BspM_Internexus	24%	396
	YP_009283122.1	AR9	24%	396
	YP_009664311.1	PBS1	24%	396
	QQO92567.1	Machias	23%	430
	QDJ97895.1	PALS_2	21%	420
	QPI17161.1	vB_StaM_SA1	20%	427
	QQO92832.1	Madawaska	20%	424
	QQM14717.1	MarsHill	20%	424

**Table 4:** **A summary of UngIn function assessment analyses and outcomes in this study.** * indicates sequences from phages encoding dUTPase enzymes. **^^^** indicates SAUGI sequences found in novel SCC-*mec* permutations.

Putative UngIn	Identification Method	Analysis	Outcome
**Ugi **	Previously identified	Library mutagenesis CS-assay	11 synthetic Ugi sequences verified as UngIns
**Ugi-2 **	Ugi BLAST	In vitro UDG assay Crystal structure	Verified as UngIn
**MCUGI1 **	SAUGI PSI-BLAST	In vitro UDG assay Crystal structure	Verified as UngIn
**MCUGI2 **	SAUGI PSI-BLAST	In vitro UDG assay	Verified as UngIn
**MBUGI1 **	SAUGI PSI-BLAST	In vitro UDG assay	Verified as UngIn
**SYUGI **	SAUGI PSI-BLAST (*Staphylococcaceae*)	In vitro UDG assay	Verified as UngIn ^^^
**JMUGI **	SAUGI PSI-BLAST (*Staphylococcaceae*)	In vitro UDG assay	Verified as UngIn ^^^
**VMY22 **	p56 PSI-BLAST	In vitro UDG assay Crystal structure	Verified as UngIn
**DK2 **	p56 PSI-BLAST (*Salasmaviridae*)	CS-assay Genomic analysis	No UngIn function *
**DK3 **	p56 PSI-BLAST (*Salasmaviridae*)	CS-assay Genomic analysis	No UngIn function *
**Goe4 **	p56 PSI-BLAST (*Salasmaviridae*)	CS-assay Genomic analysis	No UngIn function *
**MarsHill**	Synthetic Ugi PHI-BLAST (*Myoviridae*)	CS-assay	No UngIn function
**PhiR1-37 g239 **	Ugi heuristics-match (PhiR1-37 genome)	CS-assay	No UngIn function
**PhiR1-37 g280 **	Ugi heuristics-match (PhiR1-37 genome)	CS-assay	No UngIn function
**LPJP1**	Ugi heuristics-match in (LPJP1 genome)	CS-assay	No UngIn function
**S6**	Ugi heuristics-match (S6 genome)	CS-assay	No UngIn function

## Supplementary Materials

The following supporting information can be downloaded at: https://www.mdpi.com/article/10.3390/v15061348/s1.
