# Supplementary material for: A Multimodal Approach towards Genomic Identification of Protein Inhibitors of Uracil-DNA Glycosylase
Source: Viruses. 2023 Jun 10;15(6):1348. doi: 10.3390/v15061348 (PMC10303552; doi:10.3390/v15061348)
Supplement: Supplementary file 1 [file viruses-15-01348-s001.zip › viruses-2374510-supplementary.pdf]

Supplementary information

Section S1 – Methods

Protein accessions and encoding synthetic *E. coli* optimised DNA sequences

Ugi-2

Bacillus phage vB_BpuM-BpSp Gene "Bp8pS_259" product [Ugi-2] (Protein ID: ALN97938.1)

```
  1 MKFNISIISF IFTMIHKNNK RKHNLKRKDN SLMYKNIEDL NKFASKILET EISFEESITF
 61 TPDEVEENIG EKPNRDKICH STSLEDGRVI MLLTELEPNY TPWKLLELEE DGFKELYSKS
121 I
```

Codon-optimised DNA Sequence for vB_BpuM-BpSp Ugi

```
  1 ttgaaattca atatctctat tatttctttt atttttacta tgatccacaa aaacaacaag
 61 cgtaaacata atctgaagcg caaggataat tccttaatgt ataaaaacat tgaagatttg
121 aataagtttg cttctaaaat cctggaaact gaaatctcct ttgaagaaag cattaccttt
181 actcctgatg aggtagaaga aaatattggt gagaaaccta atcgtgataa gatctgtcat
241 agcacgtcct tagaagacgg tcgtgtaatt atgttactga ctgaattaga accaaactat
301 actccttgga agctgttaga attagaagaa gatggcttca aagaactgta cagcaagagc
361 atctaa
```

The annotated sequence has three potential start codons (coloured blue). Homology with PBS1 Ugi starts after the 3rd methionine, hence we cloned the sequence starting from the 3rd methionine.

SAUGI homologs

Staphylococcus aureus SAUGI protein sequence (PDB: 3WDG):

```
 1 MTLELQLKHY ITNLFNLPKD EKWECESIEE IADDILPDQY VRLGALSNKI LQTYTYYSDT
61 LHESNIYPFI LYYQKQLIAI GYIDENHDMD FLYLHNTIMP LLDQRYLLTG GQ
```

Codon-optimised DNA Sequence for SAUGI:

```
  1 atgactctgg aactgcaact caaacactat atcaccaatc tgttcaacct gccaaaggat
 61 gaaaagtggg aatgtgaatc tatcgaagaa atcgctgatg atatcctgcc tgaccaatat
121 gtacgtctcg gtgcactcag caataaaatc ctgcaaacct atacctacta ctctgatact
181 ctgcacgaaa gcaatatcta ccctttcatt ctctactatc agaaacagct catcgccatc
241 ggctatatcg atgaaaatca cgatatggat ttcctgtacc tccacaacac catcatgcca
301 ctcttggatc aacgttactt gctcacaggt ggccaataa
```

Macrococcus caseolyticus MCUGI1 protein (Accession: WP_101156358.1)

```
 1 MKQIKAHLTH YVEEILNLSS QEYLTEFIQL GIEELNWGER KIPEKLKGAI IDTYTFYNHS
61 LIKDYIYSFI GTYQGKIILL GYTKGEYEHF FYINDTDKTL HSELHLLNLT EEDLEFVNVG
```

Codon-optimised DNA Sequence for MCUGI1:

```
  1 atgaaacaaa tcaaagcgca tctcactcgc tacctcgaag aaattctgaa actctcttct
 61 caagaatacc tgactgaatt cgtacaactc ggcatcgagg aattggcatg ggtagagcgt
121 aaaattccag agaagctcaa aggtgcaatc atcgacactt acacctttta caaccactcc
181 cttatcaaag attacatcta ctctttcatc ggtacctatc aaggcaagat catcttagtg
241 ggttacacta acggtgaata cgaacatttc ttctacatca atgatacggt caagactctg
301 cacagcgagc tgcatttgct gaatcttacg gaggaggatt tggaatttgt caacgtgggt
361 taa
```

Macrococcus caseolyticus MCUGI2 protein sequence (Accession: WP_101143899.1)

```
 1 MSIKKNLTDF VERIHRLPHY HYSVEHVQLG VEEFIIEPKV ISPSLEGKVL DTYTYYSDEL
61 EDIYSFIAYY KDTVVSIGYV KGDECYSIYL NNLEETLHDE LYLINLKVED LFYANFDVG
```

Codon-optimised DNA Sequence for MCUGI2:

```
  1 atgtctatca agaagaatct gacggatttc gtagaacgta ttcaccgtct gccacattat
 61 cattattctg tcgaacatgt tcagttaggc gtcgaagaat tcatcattga accaaaggtc
121 attagccctt ccctcgaagg taaagtactg gacacctata cctactatag cgatgaactg
181 gaggatatct actcctttat agcctattac aaggataccg ttgtcagcat cggttatgtc
241 aaaggtgacg agtgctatag catctacctg aacaacctgg aagagaccct gcacgatgag
301 ctctacctga tcaacctgaa agtggaggac ctgttctatg ccaacttcga cgtgggttaa
```

Macrococcus bohemicus MBUGI protein sequence (Accession: WP_165958605.1)

```
 1 MSLSEQLCKF VERRFKYLND IWYFEHVETT LGEIFDSKDL SGDLSADKEV DTFTYFSMTL
61 DDEHVYPFIV QDDDQIIAMG YVEEEEVKLI YLTDGKSIFI DELHLLDTNK ESVQNETVG
```

Codon-optimised DNA Sequence for MBUGI:

```
  1 atgagcttgt ctgaacagct gtgtaagttt gtagaacgtc gctttaagta tctgaatgat
 61 atctggtatt tcgaacatgt agaaaccact ctgggcgaaa tctttgatag caaggatctg
121 tctggtgatc ttagcgccga caaggaagtt gataccttta cctatttttc tatgactctg
181 gatgatgaac atgtttatcc atttatcgta caggatgacg atcagattat cgcaatgggt
241 tatgtcgaag aagaagaagt gaaactgatc tatctcacag atggtaaaag cattttcatc
301 gatgagctgc atcttctcga tactaacaag gagagcgttc aaaatgaaac tgtgggttaa
```

Salinicoccus sp. YB14-2 SYUGI protein sequence (Accession: WP_052256111.1)

```
 1 MHQKLKQYIT RHLKKSEDEY LSESFVLPST ETFQSPQFQR LFDDQSLSHQ LYYSTTDDEP
61 FFPFEVYQDD TLIALGYMEE DKQHILYLKH DDEILVEEL
```

Codon-optimised DNA Sequence for SYUGI:

```
  1 atgcatcaga aattgaaaca gtatatcact cgccacctga agaaatcgga ggacgagtat
 61 ctgtcggagt ccttcgtact gccgagcacg gaaacgttcc agtctccaca gttccaacgt
121 ctctttgacg atcagtccct ctctcatcag ctgtactatt ccaccacgga tgatgagcca
181 ttcttcccgt tcgaagttta tcaagatgac actctgattg cgctcggcta tatggaagaa
241 gataaacagc atatcttgta cctgaaacat gacgatgaaa tcctggttga agaactttaa
```

Jeotgalicoccus meleagridis JMUGI protein sequence (Accession: WP_185124884.1):

```
 1 MNTKLKIYIK KYFPELSTLT WSDEAVSMSG DELFEDTKLK SLYENESLDT RLYYPIEINS
61 AILPFEIYKE ETLVALGYTN DESQKIIYFK HGAETLINHL
```

Codon-optimised DNA Sequence for JMUGI

```
  1 atgaacacca aactgaaaat ctatatcaaa aaatactttc cggagctgtc cactctcacc
 61 tggtctgatg aagcagttag catgagcgga gacgagctgt ttgaggacac gaagctgaaa
121 tctctgtacg aaaacgagag cctggacacg cgcctctatt accctattga aatcaattct
181 gcaattctgc cgtttgagat ctacaaagag gaaactttgg ttgccctggg ctataccaat
241 gacgaatccc aaaaaattat ttattttaag catggcgcgg aaactctgat caatcacctg
301 taa
```

---

p56 homologs

Bacillus phage VMY22 Gene “VMY22_4” product [p56] (Gene ID: 26625151):

```
1 MEGFKDSYTL IYVTRDEEGK MFDIKLENQT KEECEIIYGM ITDEILIWNM ILEGMF
```

Codon-optimised DNA Sequence for *E. coli* high-yield expression of VMY22 p56:

```
  1 atggaaggtt tcaaagactc ctacaccttg atctacgtga ctcgcgatga agaaggcaag
 61 atgtttgata ttaaactgga aaaccagacc aaggaagaat gcgaaattat ctatggtatg
121 attactgacg aaattcttat ctggaatatg atcctggaag gtatgttcta a
```

Bacillus phage Goe4 gene "Goe4_c00070" product (Accession: AYD87716.1). Blue coloured methionine indicates the start codon of the cloned truncated sequence

```
  1 MRKYETILIN DFMSKEIVTT VKEEDYLKVV EEKEVLENTI KMYKLEHKKI EKELKEKDEE
 61 IEKLKGNNEK WEEISLKTKQ NFTKEINKKD EEIKRKNKTI DNLMKKLIKL EEKEEQLYTL
121 KYIYDVDGVV KEYEQNGMLK EDAEELIGMD SDNWNHWSLT KEERPDKDKI VEGLLIRCES
181 NEELIKELES RNENLEIENR QLLNDRRLKI GLSDRRA
```

Codon-optimised DNA Sequence for the cloned truncated Goe4

```
  1 atgaaaaaac tgatcaaatt ggaagagaaa gaagaacaac tgtacactct caaatacatc
 61 tatgatgtcg acggtgttgt taaagaatat gaacaaaacg gtatgttgaa agaagatgct
121 gaagaactga ttggtatgga ttctgataat tggaatcact ggagcctgac taaagaagaa
181 cgtcctgata aagataaaat tgttgagggt ctcctgattc gttgtgaatc taacgaggaa
241 ctgatcaaag agttggaaag ccgcaatgaa aacctcgaaa tcgaaaatcg tcaactgttg
301 aatgatcgtc gtctcaaaat tggtctgtct gatcgccgtg cataa
```

Bacillus phage DK2 gene "DK2_00007" product (Accession: AZU99760.1). Blue coloured methionine indicates the start codon of the cloned truncated sequence

```
  1 MRKLTCNLGM KWVGEDDYLK VVEEKELLKI GQDNGIKEVC KLNRELLEQD NLMKEKDEVI
 61 ERLDKENKFH NNEFKRLSQY ILNNNYQNGK LLIVDSIIAQ CEIFDKENQG LSIRCESLEE
121 EVEGLRKENI EMIKTIKTND KKDTYTLSYS YLGSDGVTIK NYRQSGLLKE EYEEMYGMDS
181 DNWLSHSLVK DRKEVL
```

Codon-optimised DNA Sequence for the cloned truncated DK2

```
  1 atgattaaaa ccattaaaac caatgacaaa aaagatactt ataccctgag ctattcttat
 61 ctcggttctg atggtgtaac cattaaaaat tatcgtcaat ctggtctgtt gaaagaagaa
121 tatgaggaaa tgtatggtat ggattctgat aattggctgt ctcatagcct cgtaaaagat
181 cgtaaggagg tgctgtaa
```

Bacillus phage DK3 gene "DK3_00008" product (Accession: AZU99806.1). Blue coloured methionine indicates the start codon of the cloned truncated sequence

```
  1 MKEKDEEIEI LKKQWEDSPF YNWYREDNVK RMLKEKDEEI EMLKEKLDKV MNDDTYLKEI
 61 ENKNKDIDNL IEKVNKLDKE NQGLSIRCES LEEEVEGLRN QIHFCKIDEL TRYMSKNYPM
121 FAGMQVSDVV ISLLEGLKEE KEEKEEKEEK EEFWTLRYNL VVNNKEKEVV QYHMIKEDAE
181 ELIGMDSDNW NKYSLEKEVL
```

Codon-optimised DNA Sequence for the cloned truncated DK3

```
  1 atgcaagtta gcgatgttgt tattagcctc ctggaaggtc taaagaagaa aaagaagaaa
 61 aagaagaaaa aggaagaaaa agaagaattt tggactctgc gttataatct ggtcgttaac
121 aataaagaaa aagaagttgt tcaatatcac Atgatcaaag aagacgctga agaactgatt
181 ggtatggatt ccgataattg gaataaatat tctctcgaaa aggaggtgtt gtaa
```

Ung variants

SAUNG protein sequence (accession: WP_000455258.1) with hexahistidine tag:

```
  1 MEWSQIFHDI TTKHDFKAMH DFLEKEYSTA IVYPDRENIY QAFDLTPFEN IKVVILGQDP
 61 YHGPNQAHGL AFSVQPNAKF PPSLRNMYKE LADDIGCVRQ TPHLQDWARE GVLLLNTVLT
121 VRQGEANSHR DIGWETFTDE IIKAVSDYKE HVVFILWGKP AQQKIKLIDT SKHCIIKSVH
181 PSPLSAYRGF FGSKPYSKAN TYLESVGKSP INWCESEAHH HHHH
```

SAUNG-6xH DNA sequence:

1 atggaatgga gccaaatttt ccacgatatc actacgaagc acgactttaa agctatgcat

61 gacttcctgg aaaaagaata ttccaccgct attgtatatc ctgatcgcga aaacatctac

121 caggcgtttg atctgacgcc gttcgagaac attaaagtgg tcatcttagg tcaggaccca

181 tatcatggcc cgaatcaggc gcacggtctg gcctttagcg ttcagccaaa tgccaagttt

241 ccaccaagcc tgcgcaatat gtacaaagaa ctggctgatg atattggttg tgtgcgtcag

301 actccgcatc tgcaagattg ggcgcgtgaa ggtgttttac tgctgaacac tgtgctgacc

361 gtacgtcagg gtgaagcaaa ttcccaccgt gatattggct gggagacctt tacggacgaa

421 attattaaag cagtgtctga ttataaagaa catgttgtct ttattttgtg gggaaacct

481 gcgcagcaaa agatcaaact gatcgacacc tccaaacatt gcattatcaa atccgtgcac

541 ccgagccctc tgtctgcgta ccgcggcttc tttggttcca aaccgtactc taaagcgaac

601 acgtatctgg aatctgtggg caaatctccg attaactggt gcgagtctga ggcactggaa

661 caccatcatc accatcatta a

Bacillus wiedmannii Ung (BwUng) protein sequence (accession: WP_060487945.1):

1 MENVLKNDWG PLLATEFEKE YYRKLADFLK EEYSTHVVYP KVEDIFNALQ YTSYENTKVV

61 ILGQDPYHGP NQAHGLSFSV QPGVKTPPSL LNMYKELRDE YGYEIPNNGY LVKWAEQGVL

121 LLNTVLTVRQ SEANSHKGKG WEHFTDRVIE LLNEREKPVI FILWGRHAQA KKKLITNPNH

181 HIIESVHPSP LSARRGFFGS KPYSKVNTIL ANMGEREIDW EIPNL

DNA Sequence for *E. coli* high-yield expression of BwUng DNA sequence:

1 atggaaaatg ttctgaaaaa cgactgggga cccctgctgg caacggagtt cgaaaaagag

61 tattaccgca aactcgcgga tttcctcaag gaagagtaca gcacccacgt agtgtacccg

121 aaagtcgagg atatctttaa tgcgctccag tatacctcct atgaaaatac caaagtggtt

181 atcctgggcc aggacccata tcacggcccc aaccaggcgc acggacttag ctttagcgtg

241 cagccgggtg tgaaaacgcc cccgagtctt ttaaatatgt ataaagagct ccgcgacgag

301 tatggatatg agatcccgaa caacggttac ctggtgaagt gggcggagca gggcgtcctg

361 ttactaaaca cggtgctgac cgtccggcag agtgaagcga actcccacaa aggaaaaggt

421 tgggaacact tcaccgaccg agttatcgaa cttttgaatg aacgcgaaaa gccggtgatc

481 tttatcctct ggggccggca cgcccaggct aaaaagaagc tgataaccaa ccctaatcat

541 cacattattg agtctgtcca cccgtcgccc ctctccgctc ggcgtggctt ctttggcagc

601 aaaccatact ccaaagtgaa cacgatcctc gcaaacatgg gggaacgaga gatcgactgg

661 gagattccga acctgtaa

Oligonucleotides for iPCR of pRSET-C vector and SDM4_U12_Ung construct for linearisation upstream of OE-PCR/ligation cloning:

dsblntpRS_iF: `taagcttgatccggctgctaac`

LBA2delst2iR: `CATATGTATATCTCCTTCTTAAAG`

Oligonucleotides for amplifying HSV1-UNG from pTS106.1 construct:

PTrc-BE1_5p: cataacggt**tccgga**aatattctgaaatgagctgttg

rrnB-Age_3m: CATTTAT**ACCGGT**TATTGTCTCATGAGCGGATAC

N.B. *BspEI* and *AgeI* restriction sites are written in bold font.

Oligonucleotides for mutating T7 promoter to Trc promoter via inverse PCR:

T7_to_Trc_iF: `ATGTGTGGAATTGTGAGCGGATAACAAtttaactttaagaaggagatatac`

T7_to_Trc_iR: `TATACGAGCCGGATGATTAATTGTCAAatttcgcgggatcgagatc`

Sequence of Trc promoter through start codon in pBpST-CAT and sequence of T7 promoter through start codon (bases 20-102) in pRSET-C:

pBpST-CAT sequence (bases 20-102):

ttgacaattaatcatccggctcgtataatgtgtggaattgtgagcggataacaatttaactt
taagaaggagatatacatatg

pRSET-C sequence (bases 20-102):

taatacgactcactatagggagaccacaacggtttccctctagaaataattttgtttaactt
taagaaggagatatacatatg

Oligonucleotides for mutagenesis of PBS1 Ugi:

Library 1 (L1) oligos:

ugiBrndDS_F: `nnnnnnnnnnccagaagaagtagaggaagtaattg`

ugiBrndUS_R: `NNNNNNNNNNAATCACTAGTTGTTTTCCTGTTTC`

Library 2 (L2) oligos:

ugiBrndUS_R2: `ngnntcttgaatcactagttgttttcctgtttc`

ugiBrndDS_F2: `NTNCTAATGTTACCAGAAGAAGTAGAGGAAGTAATTG`

Library 3 (L3) oligos:

ugiBrndUS_R3: `nnnntcttgaatcactagttgttttcc`

ugiBrndDS_F3: `NNNCTAATGTTACCAGAAGAAGTAG`

Oligonucleotides for PCR of synthetic genes [with additional 3' homology to pRSET-C vector in Bold font]:

Ugi-2:

Bp8pS259_5p: aataaaaacattgaagatttgaataag

Bp8pS259_3m: **GTTAGCAGCCGGATCAAGCT**TAGATGCTCTTGCTGTACAG

VMY22 p56:

VMY22p56_5p: atggaaggtttcaaagactcc

VMY22p56_3m: **GTTAGCAGCCGGATCAAGCT**TAGAACATACCTTCCAGGATC

DK2 p56 homolog:

DK2p56_5p attaaaaccattaaaaccaatgac

DK2p56_3m **GTTAGCAGCCGGATCAAGC**TTACAGCACCTCCTTACGATC

DK3 p56 homolog:

DK3p56_5p: caagttagcgatgttgttattag

DK3p56_3m: **GTTAGCAGCCGGATCAAGCTT**ACAACACCTCCTTTTCGAG

Goe4 p56 homolog:

Goe4p56_5p: agcaaaaaactgatcaaattggaagag

Goe4p56_3m: **GTTAGCAGCCGGATCAAGCTTA**TGCACGGCGATCAGACAG

SAUGI:

3wdgSaUgi_5p: actctggaactgcaactcaaac

3wdgSaUgi_3m: **GTTAGCAGCCGGATCAAGCTTA**TTGGCCACCTGTGAGCAAG

MCUGI1:

McUgi1_5p: agcaaacaaatcaaagcgcatctc

McUgi1_3m: **GTTAGCAGCCGGATCAAGCTT**ACCCACGTTGACAAATTCCAAATC

MCUGI2:

McUgi2_5p: tctatcaagaagaatctgacg

McUgi2_3m: **GTTAGCAGCCGGATCAAGCTT**ACCCACGTCGAAGTTGGC

MBUGI:

MbUgi_5p: agcttgtctgaacagctgtg

MbUgi_3m: **GTTAGCAGCCGGATCAAGCTT**ACCCACAGTTTCATTTTGAAC

SYUGI:

SYUgi_5p: catcagaaattgaaacagtatatc

SYUgi_3m: **GTTAGCAGCCGGATCAAGCTT**AAGTTCTTCAACCAGGATTTC

JMUGI:

JMUGI_5p: aacaccaaactgaaaatctatatc

JMUGI_3m: **TTTCCTACGCGAATTCATGATTA**CAGGTGATTGATCAGAGTTTC

Supplementary Table S1.

Partial fractionation buffers used for small-scale growth purification.

Buffer	Composition
1	50 mM NaCl, 20 mM Tris, pH 8.0
2	100 mM NaCl, 20 mM Tris, pH 8.0
3	150 mM NaCl, 20 mM Tris, pH 8.0
4	200 mM NaCl, 20 mM Tris, pH 8.0
5	250 mM NaCl, 20 mM Tris, pH 8.0
6	300 mM NaCl, 20 mM Tris, pH 8.0
7	350 mM NaCl, 20 mM Tris, pH 8.0
8	500 mM NaCl, 20 mM Tris, pH 8.0
9	750 mM NaCl, 20 mM Tris, pH 8.0
10	1000 mM NaCl, 20 mM Tris, pH 8.0
11	1300 mM NaCl, 20 mM Tris, pH 8.0

Supplementary Table S2.

Purification buffers.

All buffers were 0.45 µm filtered prior to use.

Buffer	Composition
A	300 mM NaCl, 20 mM Tris, 20 mM Imidazole, 0.5 mM EDTA pH 8.0
B	300 mM NaCl, 20 mM Tris, 20 mM Imidazole, pH 8.0
C	300 mM NaCl, 20 mM Tris, 500 mM Imidazole, pH 8.0
D	50 mM NaCl, 20 mM Tris, pH 8.0
E	1 M NaCl, 20 mM Tris, pH 8.0
F	20 mM Tris, pH 8.0
G	200 mM NaCl, 20 mM Tris, pH 8.0

Principles of the Cellular Survival assay to assess Ung inhibition function.

CJ236 is a genomic mutant exhibiting a phenotype deficient in Ung and dUTPase activity. Due to the inability of CJ236 cells to prevent accumulation of dUTP in the nucleotide pool (*dut*⁻) or to repair uracil-DNA arising by replicative incorporation or spontaneous cytosine deamination (*Ung*⁻) a significant proportion of DNA positions will be occupied by deoxyuridine.

Schematic representation of the Cellular Survival assay for Ung inhibitory activity.

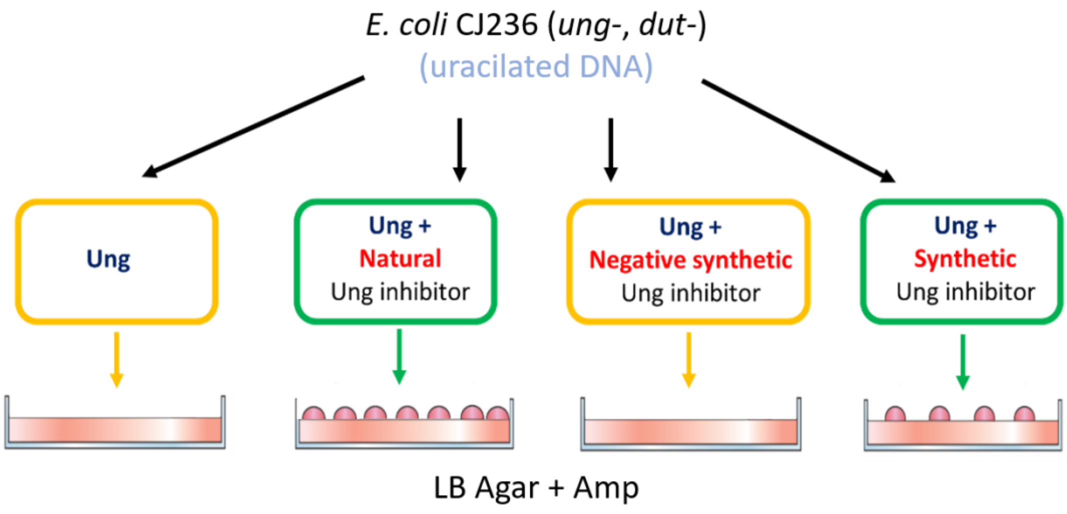

If Ung is transformed into CJ236, Ung-induced disintegration of the CJ236 genome will result, due to proximal uracil residues on both strands; this will render cells non-viable. However, transforming a natural/synthetic UngIn along with Ung protects the CJ236 genome and viable colonies can be harvested.

Supplementary information

Section S2 – Results

Supplementary Figure S1.

MSA of p56 sequences and distantly related sequences.

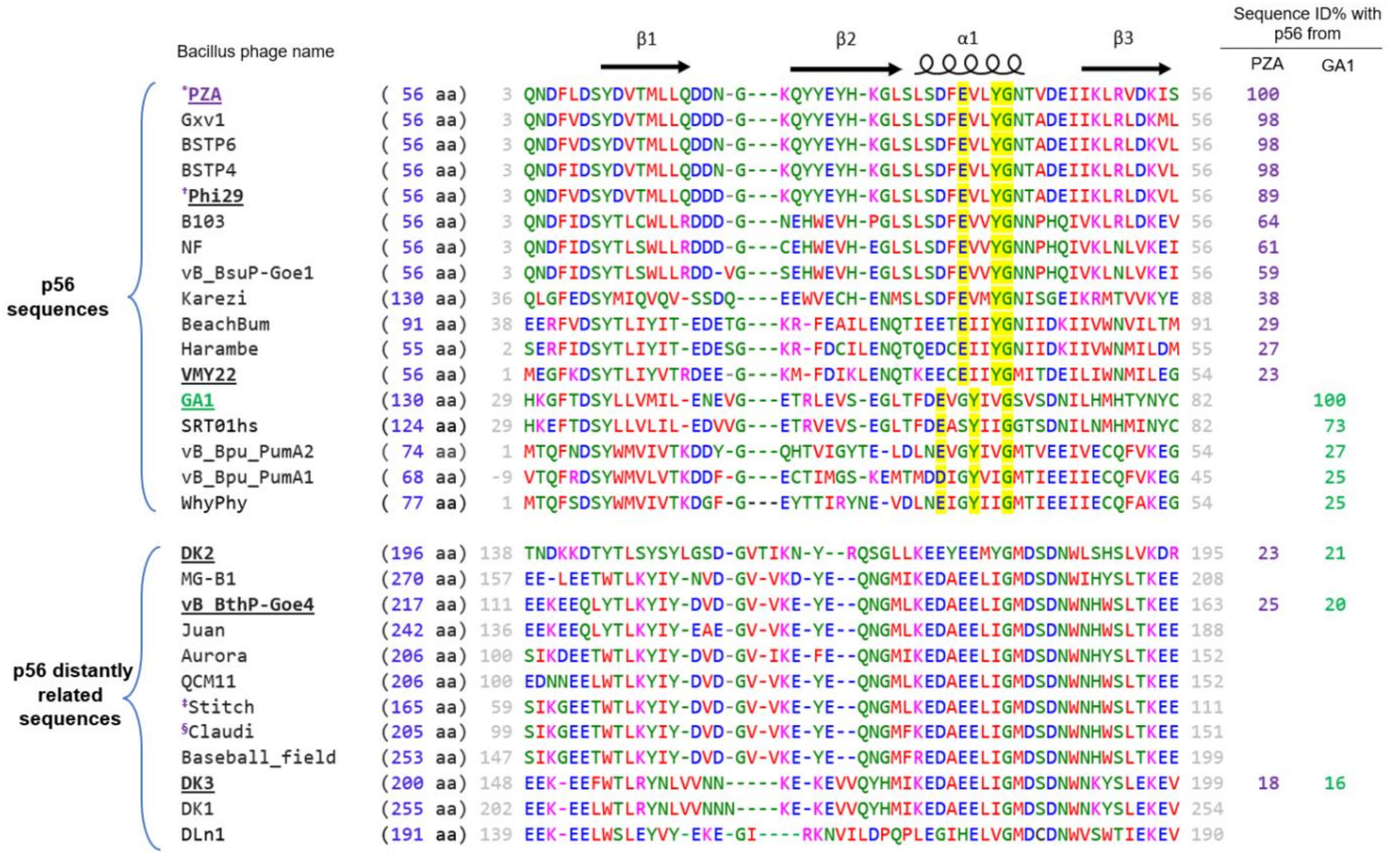

* Identical sequences are encoded by *Bacillus* phage Whiting18 (QRD99282.1) and *Bacillus* phage Arbo1 (UIS65815.1). † Identical sequence is encoded by *Bacillus* phage vB_BveP-Goe6 (ASR76788.1). ‡ Identical sequences are encoded by *Bacillus* phages StevenHerd11 (AZF88314.1) and RadRaab (ASU04169.1). § Proteins with Identical sequences in the region shown in this alignment are encoded by *Bacillus* phages VioletteMad (QDH50288.1), KonjoTrouble (ASU04129.1), and SerPounce (ARQ95541.1).

The top group of sequences shows the PHI-BLAST search generated p56 sequences using PZA p56 as an input, with the PROSITE pattern E-X(2)-Y-X(0,2)-G highlighted yellow. The secondary structure of PZA p56 is shown above its sequence. Percent identities with either PZA p56 or GA1 p56 are shown on the right. The bottom group includes sequences output from PSI-BLAST search only within *Salasmaviridae* genomes, using the VMY22 p56 sequence as input. These sequences share less sequence identity with the validated p56 sequences and their close homologs. Underlined names in *Bacillus* phage name column indicates sequences tested via an UngIn assay in this study or previous studies.

Supplementary Figure S2.

Visual U-DNA attrition assay [*Staphylococcus aureus* UNG activity] - dilution series of Ung.

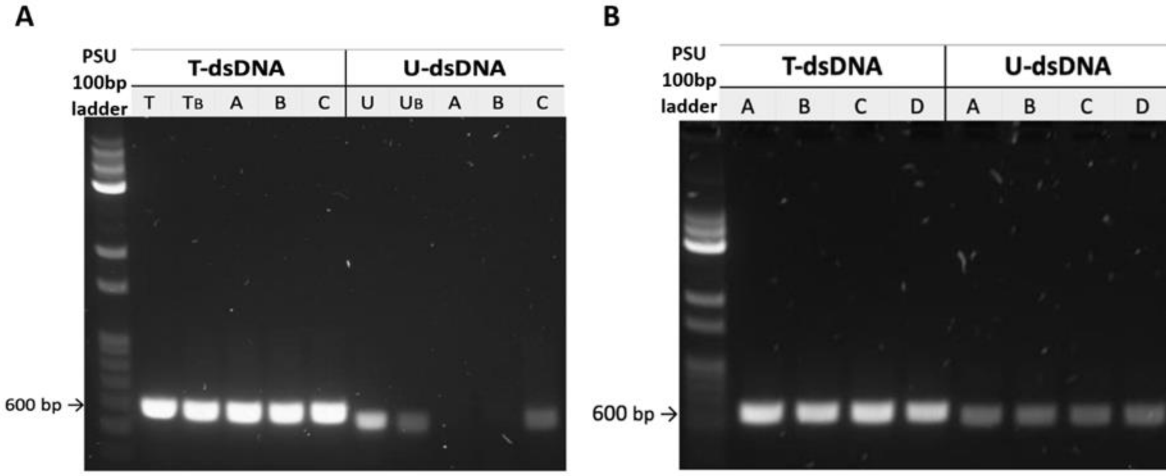

The Ung assay reaction products to verify two serial dilution of Ung from S. aureus [0.1 mg/mL, 3.97 µM] are shown on 1 % agarose gels. (A) The reaction products are split according to the DNA substrate been used: T dsDNA and U-dsDNA. In each section the first two lanes represent the controls: untreated T-dsDNA (Lane T) and untreated U-dsDNA (Lane U); and treated T dsDNA (TB) and treated U-dsDNA (UB) in the absence of Ung. (A) The first serial dilution follows the order: Ung in 1:10 dilution (Lane A), 1:100 dilution (Lane B) and 1:1000 dilution (Lane C). (B) The reaction products following the second serial dilution are displayed in the following order: 1:200 dilution (Lane A), 1:400 dilution (Lane B), 1:600 dilution (Lane C), 1:800 dilution (Lane D). The minimum concentration of Ung that showed activity on U-DNA is 39.7 nM (1:100 dilution).

Supplementary Figure S3.

Visual U-DNA attrition assay [*Staphylococcus aureus* UNG activity], validation with controls, 1% (w/v) agarose gel.

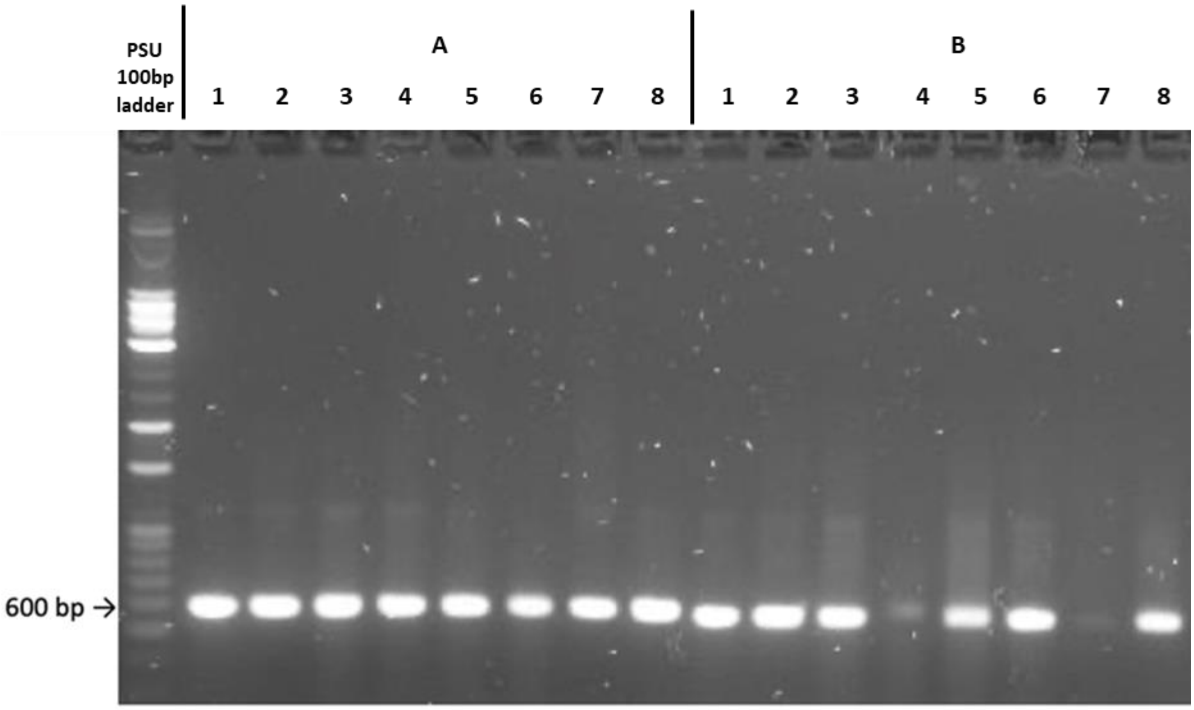

The UDG assay control is divided into two sub-sections. **(A)** T-DNA (3 µL) is used in all lanes **(B)** U-DNA (5 µL) is used in all lanes. Lanes components:

1: untreated dsDNA substrate.

2: treated dsDNA in 1X UDG buffer (NEB).

3: same as lane 2 with the addition of 1 µL *LysY/I^{Q}* cell lysate.

4: same as lane 3 plus 1 µL of Ung [39.7 nM].

5: same as lane 4 including 1 µL of PBS1 Ugi [2.6 mM].

6: same as lane 2 with the addition of 1 µL CJ236 cell lysate.

7: same as lane 2 plus 1 µL of Ung [39.7 nM].

8: same as lane 7 including 1 µL of PBS1 Ugi [2.6 mM].

Supplementary Figure S4.

Visual U-DNA attrition assay [*Staphylococcus aureus* UNG activity], to assess candidate Ung inhibitors

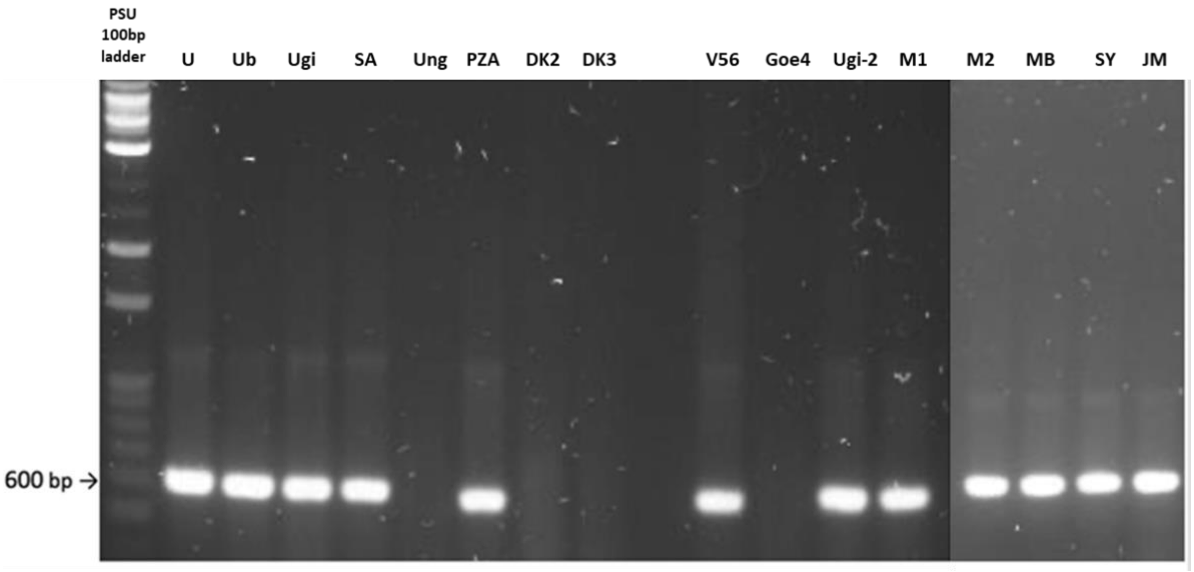

All reactions contained substrate DNA (~600 bp) in which all thymidine is supplanted by deoxyuridine. All reactions, excluding the leftmost control lane were incubated at 37 °C for 30 minutes. Lanes: (U) U-DNA substrate only; (Ub) U-DNA substrate treated with reaction buffer and conditions in the absence of Ung; all other lanes included SAUNG at 39.7 nM, and the control UngIns or the cell lysates of expressed potential Ung inhibitors as described below.

- Ugi = PBS1 Ugi
- SA = SAUGI
- Ung = Ung without added potential/actual inhibitor
- PZA = PZA p56
- DK2 = DK2 encoded potential p56
- DK3 = DK3 encoded potential p56
- V56 = VMY22 encoded p56
- Goe4 = Goe4 encoded potential p56
- Ugi-2 = Vb_BpuM-BpSp encoded Ugi
- M1 = MCUGI1
- M2 = MCUGI2
- MB = MBUGI
- SY = SYUGI
- JM = JMUGI

The UDG assay showed that Ugi-2, VMY22 p56, MCUGI1, MCUGI2, MBUGI, SYUGI, and JMUGI are Ung inhibitors while the sequences tested from DK2, DK3, Goe4 are not able to inhibit Ung.

Supplementary Figure S5.

PSI-BLAST output using PBS1 Ugi as an input – shown as MSA with query anchored dots for identity:

Query (PBS1 Ugi) 1 - 84

```
MTNLSDIIEKETGKQLVIQESILMLPEEVEEVIGNKPESDILVHTAYDESTDENVMLLTSDAPEYKPWALVIQDS
NGENKIKML
```

YP_009283008.1 1 - 84

```
...........................................................................
.........
```

QXN70328.1 1 - 84

```
........K.....E.........T...I............................................NN
.........
```

WCS68230.1 1 - 84

```
..............E.........T..........A.K................I..................NN
.........
```

ALN97938.1 51 - 112

```
--------------EISFE...TFT.D....N..E..NR.KIC.STSL.D-GRVI....ELE.N.T..K.LELEE
D
```

Detailed output sequences of PBS1 Ugi PSI-BLAST output:

>YP_009283008.1 uracil-DNA-glycosylase inhibitor [Bacillus phage AR9]

```
MTNLSDIIEKETGKQLVIQESILMLPEEVEEVIGNKPESDILVHTAYDESTDENVMLLTSDAPEYKPWALVIQDS
NGENKIKML
```

>QXN70328.1 putative uracil-DNA-glycosylase inhibitor [Bacillus phage vB_BspM_Internexus]

```
MTNLSDIIKKETGKELVIQESILMTPEEIEEVIGNKPESDILVHTAYDESTDENVMLLTSDAPEYKPWALVIQNN
NGENKIKML
```

>WCS68230.1 hypothetical protein Goe21_01200 [Bacillus phage vB_BsuM-Goe21]

```
MTNLSDIIEKETGKELVIQESILMTPEEVEEVIGNAPKSDILVHTAYDESTDENIMLLTSDAPEYKPWALVIQNN
NGENKIKML
```

>ALN97938.1 uracil-DNA glycosylase inhibitor [Bacillus phage vB_BpuM-BpSp]

```
MKFNISIISFIFTMIHKNNKRKHNLKRKDNSLMYKNIEDLNKFASKILETEISFEESITFTPDEVEENIGEKPNR
DKICHSTSLEDGRVIMLLTELEPNYTPWKLLELEEDGFKELYSKSI
```

Supplementary Figure S6.

MSA view (Flat query-anchored with dots for identity) for SAUGI homologs encoded by *Macrococcus* species.

		ID% vs MCUGI1
**MCUGI1**	MKQIKAHLTHYVEEILNLSSQEYLTEFIQLGIEELNWGERKIPEKLKGAIIDTYTFYNHSLIKDYIYSFIGTYQGKIILLGYTKGEYEHFFYINDTDKTLHSELHLLNLTEEDLEFVNVG	100%
WP_101171195.1	...........................V.....................V........Y.....................................E.......................	97%
BAI83361.1	.........R.................V.......A...N....................................	97%
WP_101144769.1	....IN.............N.I....................	97%
WP_101171460.1	...........................V.....................V........Y.........................D...........E.......................	96%
WP_101144135.1	....R......IN.............N.I....................	96%
WP_133422038.1	.........R.................V.......A...N............V.......................	96%
WP_165983527.1	.........R.................V.......A...N.D..............Y...................	95%
WP_086041446.1	.........R.L....K..........V.......A...N............V.......................	94%
**MCUGI2**	M.S..KN..DF..R.HR.PHYH.SV.HV...V..FIIEPKV.SPS.E.KVL....Y.SDE.--ED.....AY.KDTVVSI..V..DECYSI.L.NLEE...D..Y.I..KV...FYA.	

		ID% vs MBUGI
**MBUGI**	M.LS.Q.CK.VERRFKY..DI.YFEHVETTL..IFDSKD.S.DLSADKEV..F.YFSMT.DDEHV.P..VQDDDQ..AM..VEE.EVKLI.LT.GKSIFID.....DTNK.SVQNET..	100%
WP_188017758.1	M.LS.Q.CK.VERRFKY..DI.YFEHVETTL..IFDSKD.S.DLSADKEV..F.YFSMT.DDEHV.P..VQDDKQ..AM..IEE.ELKLI.LT.GKSIFID.....DTNK.SVQNET..	98%
WP_203545932.1	M.LS.Q.CK.VERRFKY.TDI.YFEHVETTL..ILDSKD.S.DLSADKEV..F.YFSMT.DDEHV.P..VQDDKQ..AM..IEE.ELKLI.LT.GKSIFVD.....DTNK.CVQNET..	95%

Three groups can be observed according to sequence similarity. Group I include MCUGI1 and 8 homologous sequences with at least 94% Identity; Group II includes one sequence, MCUGI2; and Group III include MBUGI and 2 homologous sequences with at least 95% identity. Representative sequences of these groups (MCUGI1, MCUGI2, and MBUGI) were selected to assess Ung inhibition function.

Supplementary Figure S7.

Genomic mapping of SAUGI homologues in different types of SCC*mec*.

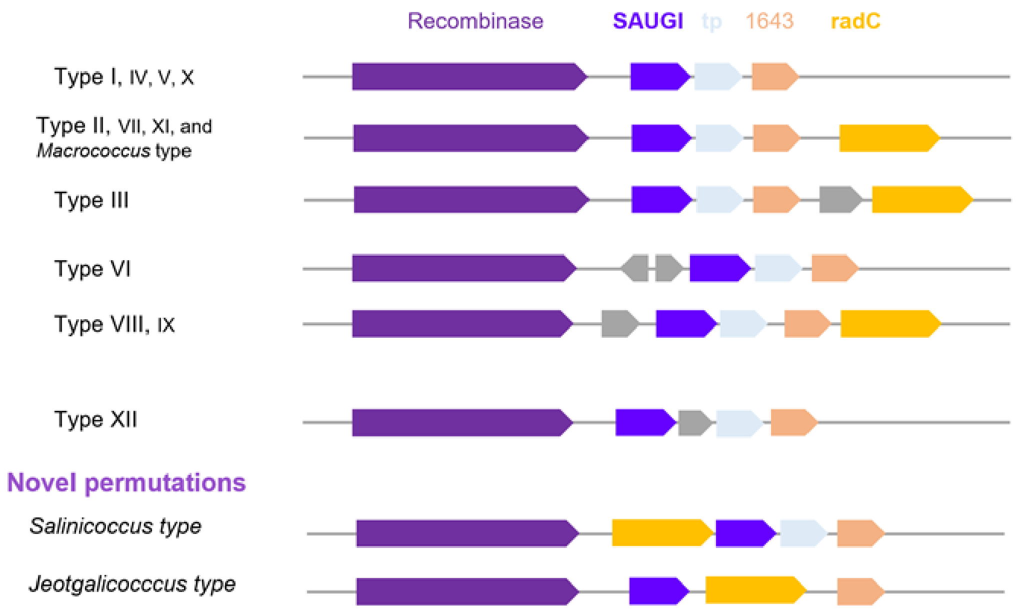

tp: a transposase usually annotated as DUF960, 1643: DUF1643, radC: DNA repair protein radC.

All SCC*mec* elements contain a *mec* gene complex (*mec*) and a cassette chromosome recombinase complex (*ccr*). Five classes of *mec* and eight types of *ccr* have been reported. Based on the combination of *mec* and *ccr*, SCC*mec* elements are classified into different types. Twelve types of SCC*mec* elements in *S. aureus* have been identified; all these types include a trio of genes: (1) *SAUGI*, usually annotated as DUF950; (2) *tp*, usually annotated as DUF960; and (3) DUF1643. This DUF 950-960-1643 trio, is always preceded by a recombinase gene and is often followed by a fourth gene, annotated as *DNA repair protein radC*. The genomic context of *MCUGI* shows similarity to known SCC*mec* types of *S. aureus*. However, the *SYUGI* and *JMUGI* genomic contexts are novel permutations in the *ccr* complex, wherein *radC* either precedes the DUF 950-960-1643 trio (in *Salinicoccus*) or separates *SAUGI* and DUF1643 (in *Jeotgalicoccus*). Gray coloured ORFs are non-conserved genes in SCC*mec*.

Supplementary Figure S8.

A comparison of Ugi variants.

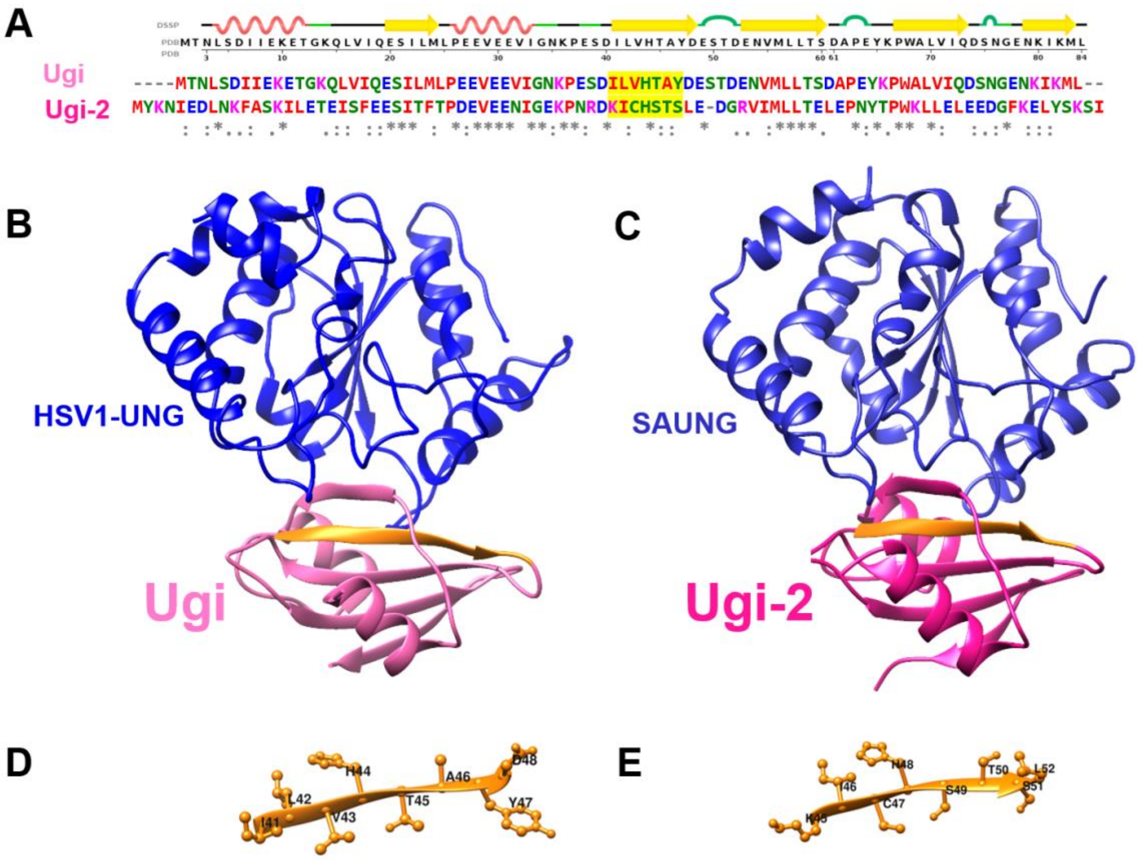

(A) structure-based sequence alignment of both Ugi variants with DSSP secondary structure of PBS1 Ugi on the top row. (B) HSV1-UNG:Ugi (PBS1) complex crystal structure (PDB: 1UDI). (C) SAUNG:Ugi-2 complex crystal structure (new data presented in this study, PDB: 8AIM). Even though Ugi variants share relatively low sequence identity (32%), their structures share the same fold, and are superimposable, with an RMSD value of 1.343 Å. (D) The 2nd β-strand of Ugi. (E) The 2nd β-strand of Ugi-2. Low sequence identity between Ugi variants can be observed in some core-forming secondary structures such as the 2nd β-strand (coloured orange in B, C, D, and E) in which only 1 out of 8 residues is identical between these Ugi variants and 4 out of 7 residues are non-conservative mutations (highlighted yellow in the alignment in panel A).

Supplementary Figure S9.

A comparison of SAUGI and MCUGI1 structures.

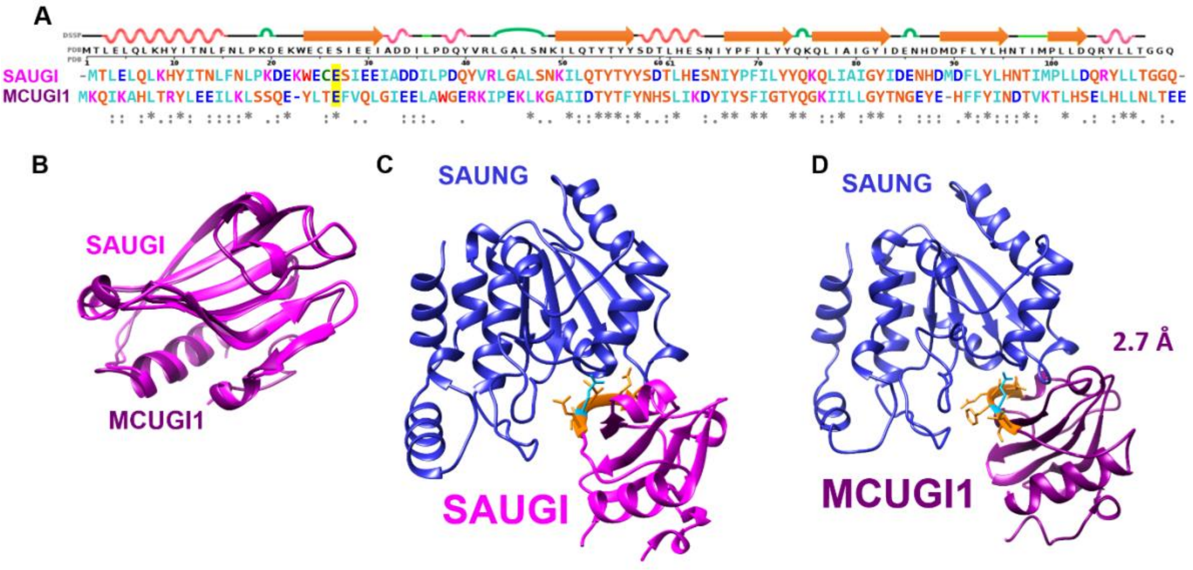

(A) structure-based sequence alignment of SAUGI and MCUGI1 with DSSP secondary structure of SAUGI on the top row. (B) Superposition of SAUGI (PDB:3WDG) and MCUGI1 (new data presented in this study); RMSD between 73 pruned atom pairs (no pair is longer than 2 Å) is 1.005 Å, and across all 93 pairs is 3.425 Å. (C) SAUNG:SAUGI complex structure (PDB:3WDG). (D) SAUNG:MCUGI1 complex crystal structure (determined in this study). Even though MCUGI1 shares low sequence identity (29%) with SAUGI, their structures are superimposable. The 1st β-strand of SAUGI and MCUGI1 are coloured orange in panels C and D. In this β-strand, only 1 out of 8 residues (a glutamic acid residue, highlighted sky blue in panel A and coloured sky blue in panels C and D) is identical between both variants.

Supplementary Figure S10.

Structure-based sequence alignment of Ugi and SAUGI.

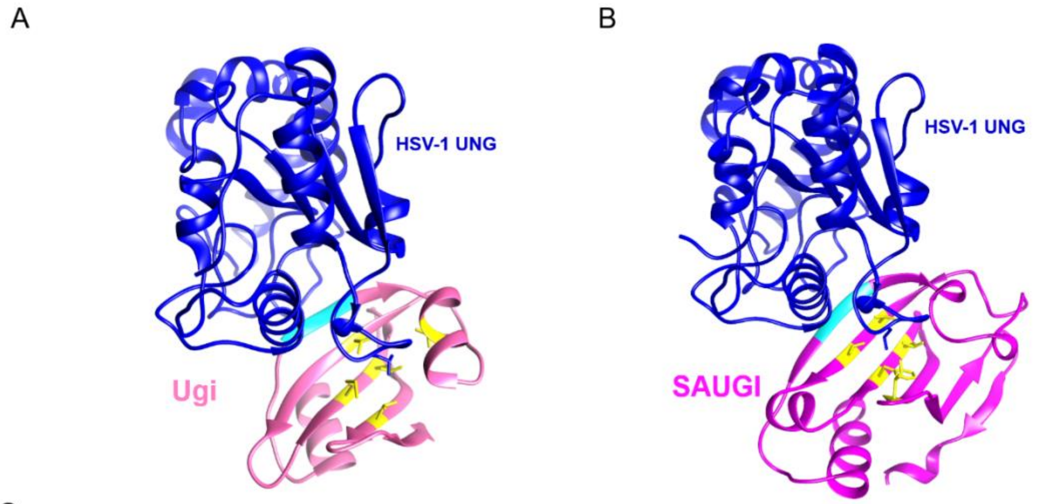

C

```
Ugi     MTNLSDIIEKETGKQL----------VIQESILMLPEEVEEVIGNKPES-------------DI
SAUGI   MTLELQLKHYIT----NLFNLPKDEKWECESIEEIADDIL---------PDQYVRLGALSNKIL
         **        *                 ***

Ugi     LVHTAYDE---STDENVMLLTSDAPEYKPWALVIQDSNGENKIKML------------------
SAUGI   QTYTYYSDTLHESNIYPFILYYQ---KQLIAIGYIDENHDMDFLYLHNTIMPLLDQRYLLTGGQ
           * *             *          *    * *       *
```

HSV1-UNG complexes with (A) Ugi (PDB: 1UDI). (B) SAUGI (PDB: 5AYS). (C) Structure-based sequence alignment of Ugi (84 aa) and SAUGI (112 aa). The apical residue [leucine in HSV1-UNG, shown as a stick] of the Ung minor groove DNA intercalation loop is sequestered by UngIn residues coloured yellow in panels A and B and highlighted yellow in panel C. Although the structures of Ugi and SAUGI share a common fold, their sequences are heterologous. These proteins share only 13 identical residues (indicated by asterisks in panel C); the only conserved motif, ESI, coloured cyan in panels A and B and highlighted cyan in panel C, is located on the 1st β-strand of each inhibitor, which docks in the Ung-DNA binding cleft.

Supplementary Figure S11.

Pairwise sequence alignment of synthetic Ugi variant (EAM-Ugi) and a phage MarsHill heuristic match (accession: QQM14549.1) generated by PHI-BLAST search using EAM-Ugi and the PROSITE pattern I-Q-E-A-M (highlighted yellow) as inputs.

```
EAM-Ugi      MTNLSDIIEKETGKQLV--IQEAMLMLPEEVEEVIGNKPES-----------DILVHTAY
MarsHill-hit MM-------KKFNQEMINKIQEAMLPL---LKEELGNEIETVEFKVEDRLAINSLFVTAQ
             *        *: .::::  ****** *   ::* :**: *:           : *. **

EAM-Ugi      DE----------STDENVMLLTSDAPEYKPWALVIQDSNGENKIKML-------------
MarsHill-hit QEMAAKLTKGGFNLDKTVFIFSDKAPEYKPVVSIYQSINKVTSLGSKRRFVKSILETIAH
             :*          . *:.*::::..****** . : *. *
```

The two proteins share 31% identity, the long insertions (highlighted green) in the MarsHill sequence take place between (rather than within) Ugi β-strands and α-helices.

Supplementary Figure S12.

Analysis of specific Ugi/SAUGI properties.

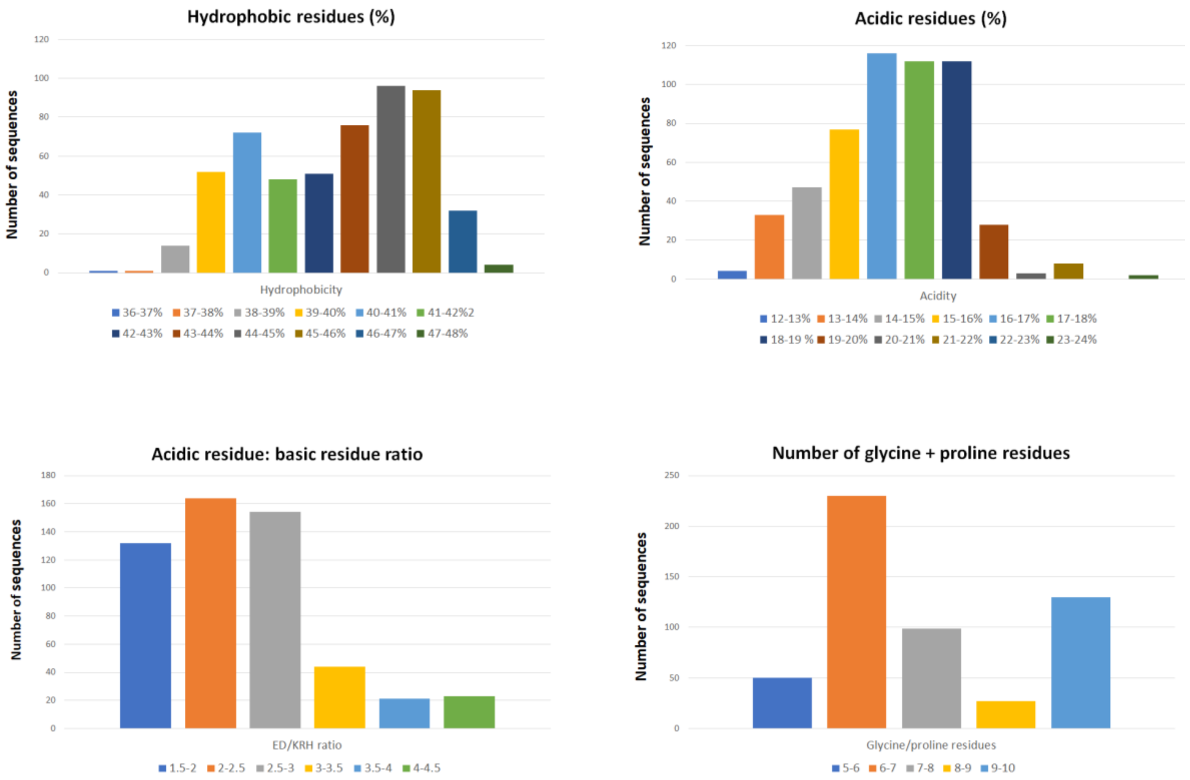

Histograms show the distribution of hydrophobicity, acidity, acidic/basic residue ratio, and glycine/proline content in Ugi/SAUGI sequences. Hydrophobic residues were defined as [IVLFMTWA], and acidic residues were defined as [ED].

Supplementary Figure S13.

Validation of filters using known Ugi-encoding genomes.

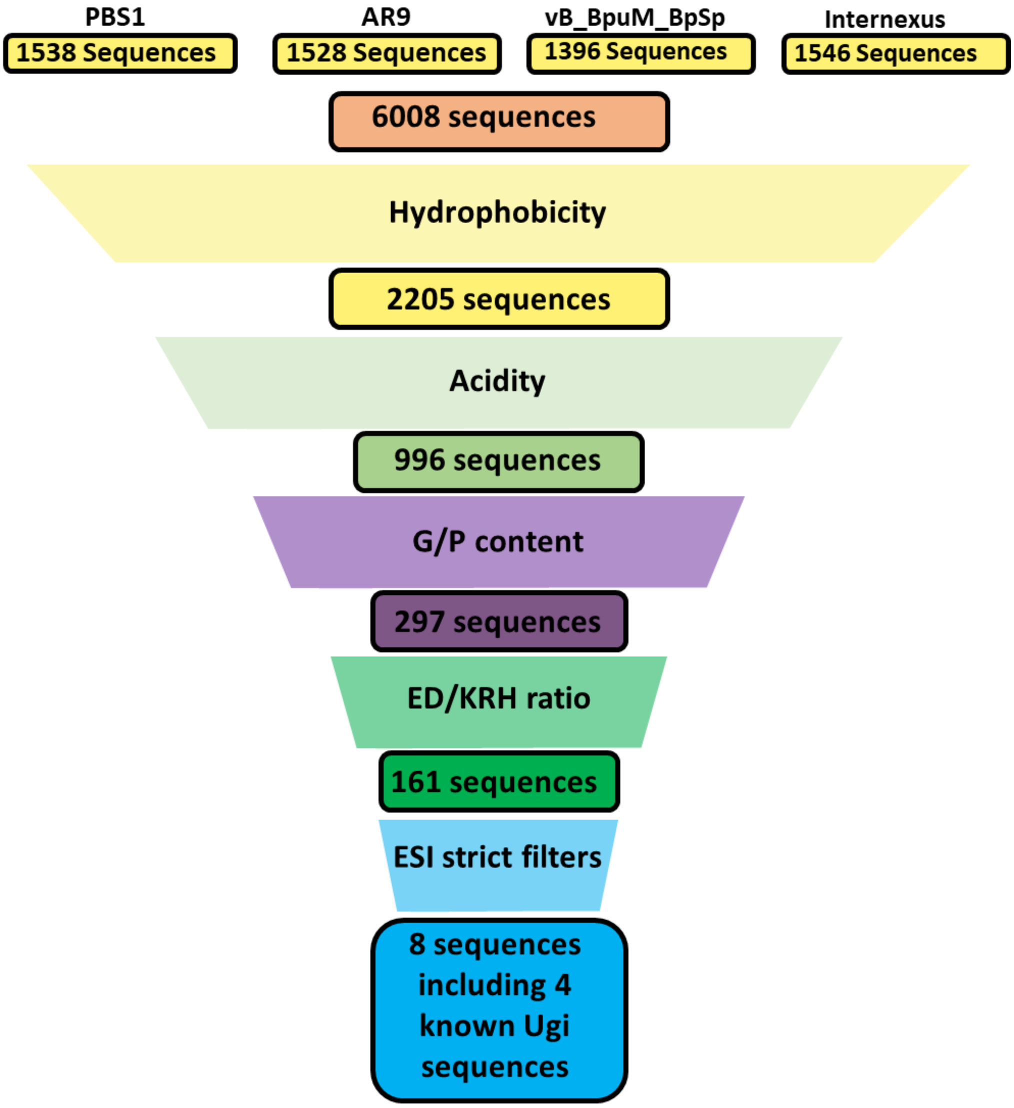

Phage genomes PBS1, AR9, vB_BpuM-BpSp, and vB_BspM_Internexus were used as inputs to the pipeline, and parameters were refined until as few sequences as possible were retrieved at final output. Several filtration steps were applied. Out of 6008 input sequences, only 8 sequences including the 4 expected Ugi sequences passed the funnel of filtration.

Supplementary Figure S14.

Schematic of the filter pipeline for the *Myoviridae* Genomes.

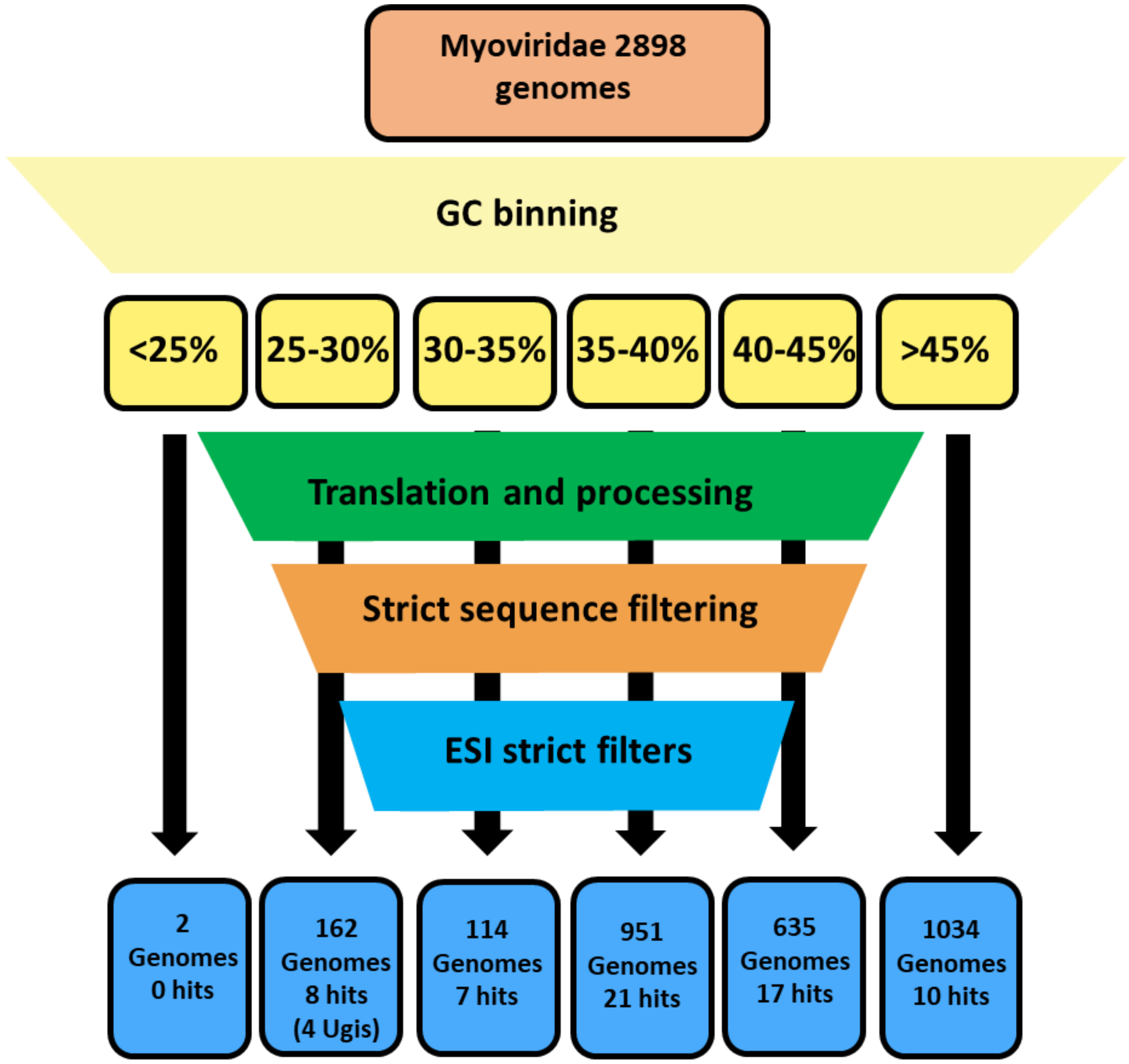

All known Ugi sequences were found in the second lowest GC content bin (25-30%). The highest heuristic matches rate (hits per 100 genomes) is found in the genomes with GC content of 25-35%.

Supplementary Figure S15.

MSAs of Ugi variants with heuristic matches from uracil-DNA phages.

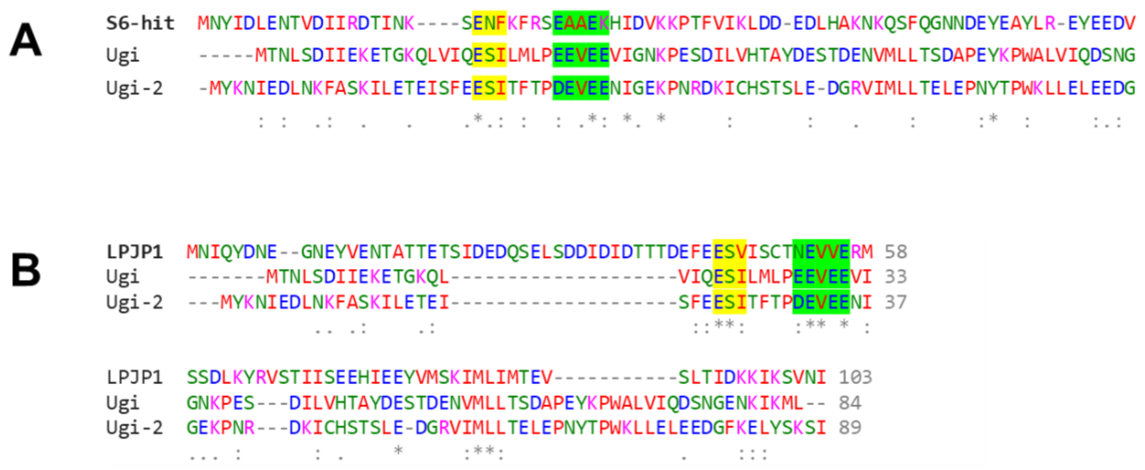

A) MSA of Ugi, Ugi-2 and a heuristic match from the *Staphylococcus* phage S6. **B)** MSA of Ugi, Ugi-2 and a heuristic match from the *Listeria* phage LPJP1. Both S6 and LPJP1 heuristic matches exhibit a plausible sequence conservation in the ESI motif (highlighted yellow) and the acidic motif located on the Ung-binding α-helix of Ugi (highlighted green).

Supplementary Figure S16.

Comparison of PZA p56 and Goe4 hit structures.

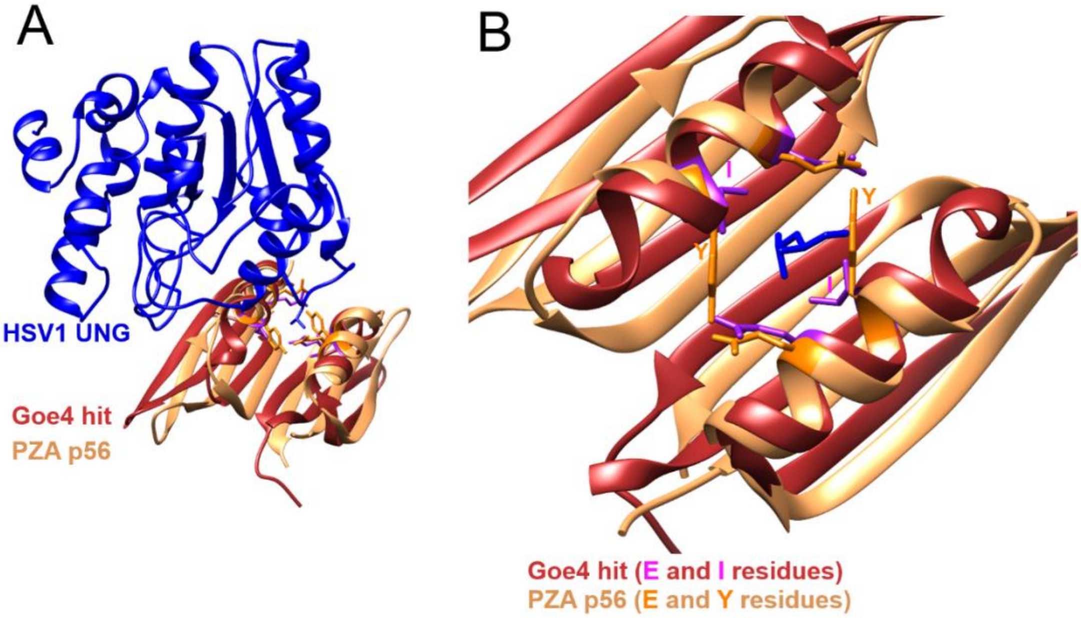

A) The structure of HSV1 UNG in complex with PZA p56 (PDB: 4L5N) is superimposed with the AlphaFold predicted structure of a hit from phage Goe4 (pLDDT score: 88.40). Monomer structure prediction of the phage Goe4 p56-homologous hit was performed, then 2 monomers were superimposed upon PZA p56 monomers. AlphaFold monomer prediction was used as it generated a prediction with high confidence score, while AlphaFold-Multimer generated a Goe4 dimerization interface (solely made by β-sheets with low confidence prediction score) that is different from the known p56 dimerization interface. Both PZA p56 crystal structure and Goe4 hit predicted structure have a similar fold, however, the recombinant Goe4 hit protein did not inhibit Ung. **B)** A zoomed overview of the PZA p56 hydrophobic pocket (orange coloured residues) and an equivalent virtual hydrophobic pocket of Goe4 hit (purple residues) showing the apical residue of Ung minor groove intercalation loop (leucine, coloured blue) and hiding Ung ribbon for clarity. E and Y residues of PZA E-X-X-Y motif are replaced by E and I in Goe4 hit. The shorter isoleucine side chain might lead to a weaker/less stable dimerization interface or may cause failure of dimerization and hence inability to inhibit Ung. Further biophysical characterisation might lead to better understanding of whether the recombinant Goe4 hit protein exists as a stable dimer, or in some other state.

Supplementary Figure S17.

Overall quality of the deposited structures

1. Ugi-2 structure in complex with SAUNG (PDB ID: 8AIM)

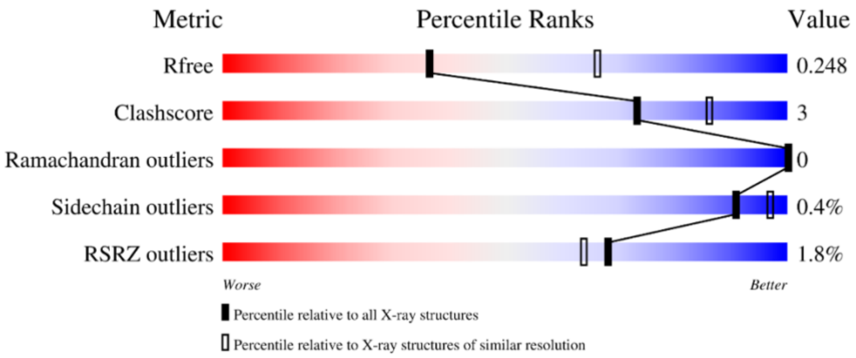

2. MCUGI1 structure in complex with SAUNG (PDB ID: 8AIN)

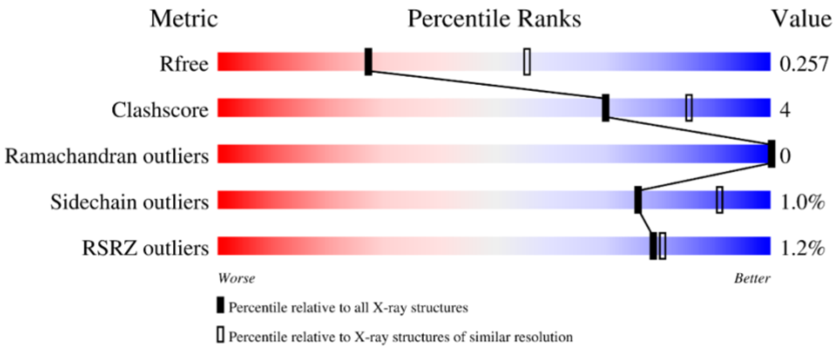

3. VMY22 p56 structure in complex with BwUng (PDB ID: 8AIL)

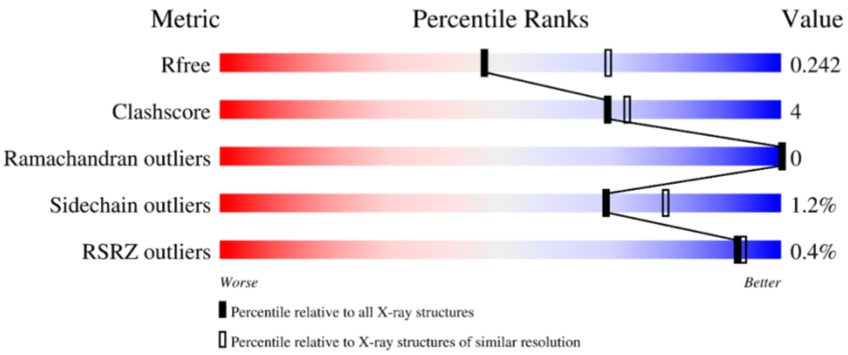

Supplementary Table S3.

Filter set and parameters used for single genome Ugi-like UngIn searches

Filter	**Parameters**
Minimum translation length	40 amino acids
Percentage of hydrophobic residues	36-48%
Percentage of acidic residues	12-24%
Number of glycine and proline residues	5-10
Ratio of acidic to basic residues	[E+D] : [K+R+H] ratio > 1.0
ESI motif filter	Lenient ESI filter: E-X-[IVLFMWPC]
ESI distance from C-terminus	50-120 residues from stop codon
ESI surrounding aspartates/glutamates	E+D count ≥ 7 in 28-residue window spanning from residue E-6 to residue E+22
ESI surrounding glycine/proline residues	G+P count= 2 or 3 in 20-residue window spanning from residue E-6 to residue E+20
ESI acidic residue position	7th or 8th residue from start of ESI motif must be E or D

Supplementary Table S4.

Filter set and parameters used for the *Myoviridae* Ugi-like UngIn searches.

These filters and parameters were run on 2898 phage genomes from the family *Myoviridae* as input.

Filter	**Parameters**
Minimum translation length	40 amino acids
Percentage of hydrophobic residues	36-48%
Percentage of acidic residues	12-24%
Number of glycine and proline residues	5-10
Acidic/basic residues ratio	[E + D] : [K + R + H] ratio > 1.0
Acidic-residue-free stretch	A maximum window of only 8 residues without a glutamate or aspartate residue
ED motifs	At least 3 [ED]-[ED] motifs
ESI motif filter	Strict ESI filter: E-[ASVFHTNI]-[LVIFMTPCW]
ESI distance from C-terminus	50-120 residues from stop codon
ESI surrounding aspartates/glutamates	E+D count ≥ 7 in 28-residue window spanning from residue E-6 to residue E+22
ESI surrounding glycine/proline residues	G+P count= 2 or 3 in 20-residue window spanning from residue E-6 to residue E+20
ESI acidic residue position	7th or 8th residue from start of ESI motif must be E or D

Supplementary Table S5.

Filter set and parameters used for the *Myoviridae* Ugi-like UngIn searches.

These filters and parameters were run on 2898 phage genomes from the family *Myoviridae* as input.

Filter	**Parameters**
Minimum translation length	40 amino acids
Percentage of hydrophobic residues	36-48%
Percentage of acidic residues	12-24%
Number of glycine and proline residues	5-10
Acidic/basic residues ratio	[E + D] : [K + R + H] ratio > 1.0
Acidic-residue-free stretch	A maximum window of only 8 residues without a glutamate or aspartate residue
ED motifs	At least 3 [ED]-[ED] motifs
ESI motif filter	Strict ESI filter: E-[ASVFHTNI]-[LVIFMTPCW]
ESI distance from C-terminus	50-120 residues from stop codon
ESI surrounding aspartates/glutamates	E+D count ≥ 7 in 28-residue window spanning from residue E-6 to residue E+22
ESI surrounding glycine/proline residues	G+P count= 2 or 3 in 20-residue window spanning from residue E-6 to residue E+20
ESI acidic residue position	7th or 8th residue from start of ESI motif must be E or D

Supplementary Table S6.

Matches generated by applying Ugi parameters set on *Myoviridae* genomes.

Matches annotated in their respective genomes as Ugi are listed in bold font.

Phage name	accession number	Heuristic match sequence
Clostridium phage JD032	MK473382.1	LVELKKEYIDKTIKLLDEVRELKVRKEVLNDEIEF LKKNEKLSSINFNELGFPVRSGNYGIDDMIINSQE KIYIKETEIEIIDSRIEMINIYTKRLSEEEQEIIS LRHFDPKINSYGEISELLMISKTVVQRKYTDALRK ITLMKYGEEAKEDRE
***Bacillus* phage vB_BpuM-BpSp**	**KT895374.1**	**VIIIEILKFNISIISFIFTMIHKNNKRKHNLKRKD NSLMYKNIEDLNKFASKILETEISFEESITFTPDE VEENIGEKPNRDKICHSTSLEDGRVIMLLTELEPN YTPWKLLELEEDGFKELYSKSI**
***Bacillus* phage AR9**	**KU878088.1**	**VNYIKIGIIERENLNWEYTSNTKIRRNFNMTNLSD IIEKETGKQLVIQESILMLPEEVEEVIGNKPESDI LVHTAYDESTDENVMLLTSDAPEYKPWALVIQDSN GENKIKML**
Clostridium phage phiC2	DQ466086.1	VHAEHIRLCSRSHLYFTSERLQNTTFFLCNCLKNK LKEETMTEYIEVGRRIFFDEEGEIIFYEGQSKGNV PERKNIKKIEYIDLEYDYVDYDKYKIIGIDIRTKQ PILEEIPVYMSEEEK
Myoviridae sp. isolate ctbc_4	MH622943.1	VNGVENMTDVYDDVEKYVKEAVSNNTEMSSSAKYC FDMAIKTRTFTNTTHSMTWLQKAMSYSVGVHHSEY KRLFENEEVIVPELDEGDVITVVPKVWWKLGFSRE KGFFSRYLVDCNKKI
Salicola phage SCTP-2	MF360958.1	LSKVYTMEEEFTSEQIPDELYDELNSSFGSVRTDL VVEYDENDPNKPQVNQNWRSFKSITTSSGDNQLVT QKAVNPETFEVEEMEGYNTFSFKVRVEFSENEDPI LRQVLGFFTFTPVNK
***Bacillus* phage vB_BspM_Internexus**	**MW749003.1**	**VNYIKIGIIERENLNWEYTSNTKIRRNFNMTNLSD IIKKETGKELVIQESILMTPEEIEEVIGNKPESDI LVHTAYDESTDENVMLLTSDAPEYKPWALVIQNNN GENKIKML**
Bacillus phage vB_BspM_Internexus	MW749003.1	VDVANLLEDTVKSGTLLNIAIWICKYNREIVTKDK TKKPCVLYVTQENSIRETLQRIFVHSTGSNIENYT EEEALRIIREEVIGIDENGEAPIDLFIKYRPNKSI STSDLDTMCDDLELEGYEVVCLIQDYTKRIRSSSY NPSDLRLELGSVVDDFTVA
Pelagibacter phage HTVC008M	KC465899.1	IKSINVQSKVTVKTRSVMTIKTKEDIIETIEYAIK DIKSGMEESGIAELEDLVKDIKDPKMMTVDLKKQY DWRTDYDWTDLNDHPVELPDGWVKV
Vibrio phage ValB1MD	MK387337.1	VKMSKEIEEAQIEEMKEVVNEESVESEGGEDTNVL PGDSQIVNIFGRKYLADSVSPRAHAIVKDLGTVDE EIKRLETSMRVAQLARSALVINFQEESKNFTEIEV QVLKK
Lake Baikal phage Baikal-20-5m-C28	MG198570.1	INVSTVISRDIRQIKLINGEELLTEVIGEDSLELF IRMPLKVVKEKVTMGEMNREANMFTNWMSFSDSED FIISRVNVLVESAVNVSVARHYLEMTENIDQDHVT RVNSNKDVPNPEKLREIARAIASQLLDDEEPTYH
Yersinia phage fHe-Yen9-01	KY593455.1	IQRSKMQEGKFYSFNEAFRADFIEDNSTNENMLRL IEEGGGSFEVLEMVTEDTGKYVRRVRMKNGEVYDA DIPGDEYFELSNWEFKYFVEVVGLTTEGAQSMSLV VTRENAEEMIELIKKAFNK
Proteus phage SJ_PmiM	MW367898.1	LFLILIGMCAFNYRKNLMFLMNREEALEAMKDGHK IMHEYFSPEEHFYMVNGNIIDESGYDWNFLFFKRD MYEKGWAIKE

Providencia phage PSTRCR_127	MW358927.1	INNITSLVNRLFDNGKTPMEMFGVNWNQIEELEKT METIQFECVEKPDDVDWYTKGKIYDAVNSRVNSIC EDHYVKTDDGVLGLISNVNGVYMSLLIHDIKFRRV
Providencia phage PSTRCR_121	MW385300.1	INNITSLVNRLFDNGKTPMEMFGVNWNQIEELEKM METIQFECVEKPDDVDWYTKGKIYDVVTSRVNSIC EDHYVKTDDGVLGLISNVNGVYMSLLIHDIKFRRV
Cronobacter phage vB_CsaM_GAP32	JN882285.1	IMRYEAFMGTDEFLYSGESGEISTKMLKFMEAITD ENEAGTFEIKEEVVGGIPLYYASVCFPDWAEALFL IQFYEYSWELLEES
Cronobacter phage vB_CsaM_GAP32	JN882285.1	VFVCQFTTTKIRINYMSKVLKHVSLVTENCEVFTL NGSDLIWTDYQKQDDIRNPFERSMSEVLGLCFSKD SQILLEKSFISSLQFNKRTDITWVEFVFDNDETEI VTICWPEGEENRYLTEHPGQRWFVTPEGNFMFQSW YATDKERMINLDESIYDLRAMDKKA
Vibrio phage 1.193.O._10	MG592562.1	MEYFHFKKVNDLRAFLAKRHEDINLNASISQPSFY FVTTDKVVHRATGTKMLAQVLNDLFGNYYSVEDSV TVTRQRGTLAYALQEKQEKVETPVQEEKEEVITEP EVEEVVEDSPSLISLEVEEDVTEDSKVPDWAWIES LENTPEDKLELDKYAESEFSVKLSRTMKLGNMVKK FKEELAKR
Vibrio phage 1.187.O._10	MG592553.1	MEYFHFKKVNDLRAFLAKRHEDINLNASISQPSFY FVTTDKVVHRATGTKMLAQVLNDLLGNYYSVEDSV TVTRQRGTLAYALQEKQEKVETPVQEEKEEEEVIT EPEVEEVVEDSPSLISLDVEEDVVAEGSKEPDWAW IESLENTKDDKNEFDKYCESEFSVKLSRTMKLSNM IKKFKEELAKR
Acinetobacter phage Ab_121	MT623546.1	VDFDSINQQIENFIESRVNEFKGCDPSALMDIFEN PIYAEKLEEFMEKCGPDIDDDIFAKRINQHFKEIG FETEVNVGKIFDEALCIQLVMQPFFVNALKEFVGY QK
Synechococcus phage B3	MN695334.1	IKTLNKTLKNPTQMKISRKSIIDDRSAIGHFILSA ITEVRPEVFETIDRGDDEFEVCMTFNGVEIPIDVV INKWVERNRQGINKAAVNLIAEKLDVINDKIRQAE EEIQEKLSDLKSDIAESFNLQYNSWDDYFYENDDH A
Vibrio phage RYC	AP014858.1	MAQRLFFRNKALLVNALKEYFPTLDVEKSKSRIRS RFSLVFGEEVIYSGTVSEMVSNLNKVAGKDIFVKR AYLKGKSGYVIFLNEDPKELVVEDAVQEQEEKSEV EKTEIDKEVVAPDAVDWEWVEGLGNTKEDKLALDQ YAEKFDVKLSRTMKIENMVAKFKEALEAK
Marine virus AG-345-E15 Ga0172270_11	MH319741.1	IRDLESEIQKLTEQLADRNTEHEKLTTFKDKLSST YEELSSRKDTISYYDFMYSLLRDGGVKTKIIKKYL PLINQQVNRYLQKMDFYINFTLDEEFNETVQSPIH EDFSYASFSEGEKMRIDLALLFTWREVARMKNSVN TNLLIMDEVFDSSLDGMGTDEFLKIIRYVIKDTNI FVISHKPEMHEKFESMIRFEKVKGFSRMVEQ
Aeromonas phage Aszh-1	MN871442.1	MKMFSTVTSLLTVRNIKFFEFYIKDISSGELIWFT YDGFAYLFKKDTNEFIDCEIDYDDPEEPQRVVDKF INSPCDLPHRFSLVDQIDQLQEELKDRLYQDFRFN RDMTDKK
Bacillus phage Shbh1	KU640380.1	IRRMMKVHEISLVKYAGEYWVTLADFSHTRDVSNY ADHASVKSAIRTFVVRNNPDKYISFRGEQQIKNLI TENKTNNLFILPHFQGHTRTALIHWSILEELTDKF PTLEEYEEDFENFIEEAKEFMDQPKPVQDPESSVI GERAAVIHSLRTQIQRLDEEIKVRQSNREKILQAI NALENLDVTEV

Pseudoalteromonas phage HM1	KF302034.1	IMSDNTLQKIRNLEQDIVDLQQLLRVESSFRSKYELEVDAIYKTLIKMGMLETFKEEHFSGGREDDVEEFTHFMGESPLLGEDISVNMSGYTLEEHKQRVAALREFNEGE
Escherichia phage vB_EcoM_G8	MK373787.1	IMDFFTPEANQKNINKFFSIASTITRQLETALLCMETVENIHTYPFKNICGWEGYKIVISLREVKCAYSPTDKEIYQQKCDEIVNTPKEETTLEELMECLDDSPEPVEIRPEVIALEKAYKEVLEISNKAQKEYEQAKKIWEESVNRLDRLEQALQLIK
Synechococcus phage B23	MN695335.1	IKTLNKTLKNPTQMKISRKSIIDDRSAIGHFILSAITEVRPEVYETIDRGDDEFEVCMTFNGVEIPIDVVINKWVERNRQGINKAAVNLIAEKLDELHVKILQAEQEIQEKLSDLKSDIAESFNLQYNSWDDYFYENDDHA
Aeromonas phage AsSzw2	MN871441.1	MKMFSTVTSLLTVRNIKFFEFYIKDISSGELSWFTYDGFAYLFKKDTNEFIDCEIDYDDPEEPQRVVDKFINSPCDLPHRFSLVDQIDQLQEELKDRLYQDFRFNRDMTDKK
Acinetobacter phage TAC1	MK170160.1	VDFDFINQQIENFIESRVNEFKGCDPSALMDIFENPIYAEKLEEFMEKCGPDIDDDIFAKRINQHFKEIGFETEVNVGKIFDEALCIQLVMQPFFVNALKEFVGYQK
Escherichia phage T2	LC348380.1	MNLIKIKQLFVNYEFFTPETNQKNINKFFSIASTITRQLETALLCMETVENIHTYPFKNICGWEGYKIVVSLREVKCAYSPTDKEIYQQKCDEIVNTPKEETTLEELMECLDDSPEPVEIRPEVIALEKAYKEVLEISNKAQKEYEQAKRIWEESVNRLDRLEQALQLIK
Aeromonas phage CC2	JX123262.1	MKMFSTVTSLLTVRNINFFEFYIKEISSGELSWFTYDGFGYLFKKDTNEFVDCEIDYEDPEEPQQVVDKFINSPCDLPHRFSLVDQINQLQEELKDRLYQDFRFNRDMTDKK
Aeromonas phage AS-yj	MF498774.1	IMTDVMRIRFLSEKDKEHFVSRSVNANTHIANHMGMVWNRVSFDGRRWYLVDENNNEVVVDDGDVDSFIHPSEYQFFEWDILPIEKKKSIKELWDIAQQKKAEYDDAMTEYNKAVMEKLDESAI
Vibrio phage 1.161.O._10	MG592529.1	MEHFHFKKINDLRAFLSKRHEEINLNASISQPSFYFVTKDGKVHRATGTKMLAQVLNELFGNFYSVEDSITVTRQRGTLAYTLQEQEKVVKAEVVVPVEEVKDVVSEPEVLEELAVDESVAEVAVEEVVEDAKEPDWAWIESLENTKEDRIELDRYAEEEFSVKLSRTMKLANMVKKFKEELAKR
Aeromonas phage AS-szw	MF498773.1	IMTDIMRIRFLSEKDKEHFVSRSVNANTHIANHMGMDWNRVSFDGRRWYLVDENNNEVVVDDGDVDSFIHPSEYQFFEWDILPVEKKKPIKELWDIAQQKKAEYDDAMTEYNKAVMEKLDESAI
Myoviridae sp. isolate 131	MN856013.1	MKYPEYSGTYYDKFKVTEIDYGVVTVKFFSELHEDWIEAWAQTEVEEITLHVGDSLRPEHDHDEDFYIGQLKDNVAWELEASSIDQEFGSKFIFNHHIQQINQVLQDDFADKL
Campylobacter phage C2	MG065655.1	VSDMAKIRKLSSNQVVKDFESGEILYVRDADDEGEDLVMLVGHAEDGCIQAVFLETAMVHWIELDLKARKPKAEILIYED
Synechococcus phage ACG-2014d isolate Syn7803C45	KJ019028.1	MKHHIPDEIRANCFSCFTSLNAAERACVLLGDEAYRESLDLENDDAPCWQIPSGEHSTFAGWNPQCVPTIEYIVWKLKNREGIIKGEIY
Escherichia phage APCEc02	KR698074.1	VSDMAKIRKLSNNQVVKDFESGEILYVRDADDEGEDLVMLVGHAEDGCIQAVFLETAMVHWIELDLKARKPKAEILIYED

Salmonella phage STML-13-1	JX181828.1	IMSVKMKGVEFNAYYNDDEYWVKNAWHDDHCVKVN<br>GEYREELDENIPDDADVVIESGTVYIPVEGENGAE<br>EKDISLVNHFKTWRKQKNFSFIVVTVKKDKLAEVR<br>EAMRCIPGVIEVKGN
Escherichia coli O157 typing phage 14	KP869112.1	VSNMAKIRKLSNNQVVKDFESGEILYVRDADDEGE<br>DLVMLVGHAEDGCIQAVFLETAMVHWIELDLKARK<br>PKAEILIYED
Escherichia phage naswa	MN850595.1	VSNMAKIRKLSNNQVVKDFEPGEILYVRDADDEGE<br>DLVMLLGHAAEDGCIQAVFLETAMVHWIELDMKAR<br>KPKAEILIYED
Escherichia phage V18	KY683736.1	VSNMAKIRKLSNKQVVKDFEPGEILYVRDADDEGE<br>DLVMLLGHAAEDGCIQAVFLETAMVHWIELDMKAR<br>KPKAEILIYED
Serratia phage Muldoon	MN095771.1	MKNQNSVRVFTPNTYVLCMEFFYGDDDFQHNHRCT<br>FDPKKYSLDMLREFVTDAKEVTDEQPEELPEWFTK<br>KWDHLVQLLKSCEVWWTLAYVDVWYVDEVGAPWSL<br>EKL
Enterobacteria phage vB_EcoM-FV3	JQ031132.1	VSNMARIRKLSNNQTVKDFEPGEILYVHDADDEGE<br>DLVMLLGHAAEDGCIQAVFLETAMVHWIELDLKAR<br>KPKAEILIYED
Escherichia phage naam	MN850630.1	VSDMAKIRKLSNNQIVKDFEPGEILYVRDADDEGE<br>DLVMLLGHAAEDGCIQAVFLETAMVHWIELDMKAR<br>KPKAEILIYED
Campylobacter phage D#	MG065647.1	VSDMAKIRKLSSNQVVKDFESGEILYVRDADDEGE<br>DLVMLVGHAEDGCIQAVFLETTMVHWIELDLKARK<br>PKAEILIYED
Serratia phage PS2	KJ025957.1	MITANAVKVFTPNTYVLNLDFFWGDGDICTGYRKI<br>LDPKCFDINQIREFLIEAKEVTEEQPEDLPEWFTD<br>KWPHIYTLLKSDEDIWWTLERADIWYVDEVGTPWS<br>LEKI
Vibrio phage 1.031.O._10	MG592415.1	IMATTGAKLLEICEAILTNPDYEGERLSDESAIQV<br>TLGKEDVKSFSKLAQEAGLTTKTVGDDTVRVYVDA<br>DTADDSMKAINAIDRAQSYKLEARPVNLEGWHAEP<br>GE
Aeromonas phage asfd_1h	MN871507.1	MEHVKYRFKEYSHISDFVHKDNVNLAIYRDLHDKE<br>FYLKQVEVDKYVAVDAIGDRMYDENIFDFNKTEVD<br>EFLEIVDELAAKPVEKPAEEPVEKPLTISQLHDWS<br>IRHALTSLSVEKVVEMYKIYIK
Escherichia phage nomo	MN850578.1	VSDMAKIRKLSNNQVVKDFEPGEILYVRDADDEGE<br>DLVMLLGHAAEDGCIQAVFLETAMVHWIELDMKAR<br>KPKAEILIYED
Escherichia phage PDX	MG963916.1	VSDMAKIRKLSSNQVVKDFEPGEILYVSYADDEGE<br>DLVMLVGHAEDGCIQAVFLETAMVHWIELDLKARK<br>PKAELIYED
Salmonella phage ISTP3	MT974436.1	IMSVKMKGVEFNAYYNDDEYWEKGAWHDDHCVKVN<br>GEYREELDENIPDDADVVIESGTVYIPVAGESGAE<br>EKDIQLVTHFKNWRKKKNFSFIVVIVKKDKASEVR<br>EALKNISGVMEVKGN
Pectobacterium phage DU_PP_I	MF979560.1	LMSKNTRYSHLLFHIMSPELREQFFTDDEENFDAG<br>GDLFETLHPEKAEVLVSLLEPHLEYVIKELKFQRD<br>HNILLGKGDELGAARLAICHRADKLDW
Halorubrum coriense virus Hardycor2	MN901520.1	MARSGRGRTVTNGRIEVVTEPEDSDFEFPPLFQEQ<br>SFHYQETDFYRTVDGQLFHYITLRNDEDFNWPPTV<br>DVGINIIEVNDDTHHS
Cronobacter phage CR3	JQ691612.1	LMSKNTRYSHLLFHIMSPELREQFFTDDEENFDAG<br>WDLFETLHPEKAEVLVSLLEPHLEYVIKELKFQRD<br>HNILLGKGDELGAARLAICHRADKLDW

Vibrio phage vB_ValM-yong1	MN563793.1	MSELHQKAEEFVLCALADDTYGDSEQGNEVGLFLL<br>QLILRHKKGQLRAAQMDELGVKTMPIVVSGDLGCQ<br>EITPL
Bacteriophage P27	AJ298298.1	LYSRTYKSAEEAMKKFENITVLHVDDFDYTNPELL<br>PEVVKAIDVADIVIRGKRIVKNRLACTSGAMTETT<br>SQQDDYEGICLEPDSFAVNVYHLLHATQVLHMSSN<br>HETKTLGSEILNFACEYAKSAAEKELAQ
Klebsiella phage vB_KaeM_KaOmega	MN013077.1	LMGKYTRYSNLLVHVMSPELREEFFGDEEEDTDGG<br>WDLFETLHPEKAEMLMEVLQPMLDDTIKELQFRRD<br>YNTLLGQGKQTEATRLMICHRASKINWDED
Rhizobium phage RHEph04	JX483876.1	MAKFNKFRKGASTFVCECCGHHTRETGQALGAKIC<br>YACFELAGLENMLSDDGEEQFAKVGADEVKSWMNE<br>IRKRSEAEFERAKASFSSLAPYFPSDEDFTTEAPL<br>LSF
Escherichia phage ECML-4	JX128257.1	IMSVKMKGVEFNAYYNDDEYWVKNAWHDDHCVKVN<br>GEYREELDENIPDDADVVIESGTVYIPVEGENGAE<br>EKDISLVNHFKTWRKQKNFSFIVVTVKKDKLAEVR<br>EAMRCIPGVIEVKGN
Halorubrum coriense virus Serpecor1	MN901521.1	LSLRVLEEHKTMTELTNFTDPTPDLTDHERRLLRW<br>VGADERLIEVCSFDVTSMERKRGDGKAVTRNSALV<br>EVKKYTREWESLTEENAEDFDHYGGHFFSALWDGD<br>LYEAYTRADYNNKAIMLEVFDVRRINSTRPAHAAE<br>VTV
Salmonella phage GEC_vB_MG	MW006477.1	VKMTKDLWEVFQDDDEIKVIVSGSLEEGCGWRSYS<br>DVCSEINTLQDAKLIAAAPELLDAVLDLKHKLYGN<br>GPANPKIEELLNRLKGE
